# Influence of musical context on sensorimotor synchronization in classical ballet solo dance

**DOI:** 10.1371/journal.pone.0284387

**Published:** 2023-04-18

**Authors:** Mohammad Talebi, Adriaan Campo, Noelle Aarts, Marc Leman

**Affiliations:** 1 Department of Science, Institute for Science in Society (ISiS), Radboud University, Nijmegen, Netherlands; 2 Department of Musicology, Institute for Psychoacoustics and Electronic Music (IPEM), Ghent University, Ghent, Belgium; University of Illinois at Urbana-Champaign, UNITED STATES

## Abstract

Several studies have addressed motor coordination in dance, but few have addressed the influence of musical context on micro-timing during sensorimotor synchronization (SMS) in classical ballet. In this study, we analyze the Promenade in Arabesque of the Odile variations, first as a dance-music fragment non-embedded in a musical context, then as a dance-music fragment embedded in a musical context at two different instances. Given the musical structure of the fragments, there are repeats of patterns between and within the fragments. Four dancers were invited to perform the three fragments in twelve successive performances. The beats of the music were extracted and compared with the timing of the dancers’ heel movements, using circular-linear smooth regression modelling, and circular statistics. The results reveal an effect of repeat within fragments, and an effect of musical context between fragments, on micro-timing anticipation in SMS. The methodology offers a framework for future work on dynamical aspects of SMS.

## Introduction

Sensorimotor synchronization (SMS) refers to the coordination of rhythmic movement with an external rhythm, and it can involve a simple tap of the finger, a metronome, or whole-body dancing to music [1, 2]. Musical rhythms usually span multiple metric levels, offering different plausible tempi for human synchronization. The most plausible and distinct periodic pulse to which most humans synchronize, is called the beat. Its frequency has been related to a 2 Hz human body resonance phenomenon [3, 4].

SMS to a beat has received a lot of attention in various empirical studies and disciplines (for an extensive review, see e.g., [1, 2, 5, 6]). Previous research has covered metronomes [7, 8] and music [9, 10], in motor tasks related to finger tapping [10, 11], walking and running [12–14], and dancing [4] among others. To evaluate SMS, synchrony and asynchrony have been assessed as the difference in the mean delay (or the accuracy) between a stimulus (typically a beat onset) and a subject’s corresponding movement (typically a movement onset), and as the variability in the mean delay over time (or the precision). It has been assumed that SMS is based on timing predictions, for which automated error correction mechanisms of phase and period, continuous or discrete, have been identified [1]. SMS, moreover, has an anticipatory component, which is manifested in a negative mean asynchrony (NMA), meaning that the movement comes before the audio beat. Music training [15] and dance [16–18] training have been reported to reduce NMA.

However, few or no attention has been devoted to the fact that a beat can be embedded in a musical context that possibly influences the micro-timing of SMS. This would imply that sensorimotor prediction mechanisms of SMS can be influenced by cognitive processing of music. An example of change in musical context while the tempo, and the beat, remain constant is when patterns, such as musical and harmonic phrases, are repeated. In such contexts, SMS would be induced by the beat, but the micro-timing of SMS might be influenced by pattern repeats.

Although pattern repetitions are common in music, their influence on SMS has hardly been studied. Instead, attention is focused on other aspects that could affect SMS. In general, findings on musicians are extrapolated to dancers, suggesting that dancers are better than non-dancers at synchronizing to the rhythm [18], especially in tasks involving whole-body synchronization [4, 8, 16, 17, 19–21]. When dancers are asked to bounce to the beat by bending at the knees, dancers’ synchronization is more precise and more accurate than non-dancers [8, 19, 20]. Similarly, dancers show lower variability in leg movements during a dance synchronization task [22] and better coordination with perceived dance movements in the presence or absence of auditory cues or music [23]. Improved synchronization accuracy may be influenced by dancers’ better proprioception [24], better postural control [25], greater stability, and stronger coupling between limbs [26]. In musicians, SMS accuracy and precision seem to be significantly improved only after considerable training [1], and there are several indications that a high level of dance training also positively affects dancers’ timing capabilities [16–18]. An exhaustive review of the different context factors affecting SMS is beyond the scope of this work. Previous research has indicated an influence of health [27], cultural background [28], physiology and handedness [29, 30], prior training and experience [17, 31], age [32], tempo [33], expressivity [34], aesthetics [35], amongst many others. When it comes to SMS in dance studies, researchers have investigated the influence of the type of stimulus [36], tempo [37], and level of training [16, 17].

The overall picture emerging from the literature is that SMS might be part of a larger phenomenon of synchronization-based interaction, which influences bodily articulations and phenomena known as entrainment, spatiotemporal movement, expressive timing, and arousal. Van Dyck et al. [38] examined the effect of the sound pressure level of the bass drum on contemporary dance movements, showing that the louder sound of the bass drum facilitated rhythm and tempo perception of the music, increased motor activity, and influenced the dancers’ entrainment speed. In other research, music with a distinct beat (or low "fluctuation entropy") seemed to increase dancers’ local movement, whereas music with a strong rhythm (or high "low frequency variation") made the dancers move more slowly and more on the spot [39]. In yet another case, specific movement patterns were linked to musical characteristics and even to character traits, e.g., [40–42].

Humans are highly sensitive to subtle changes in timing, in both dance [35] and music [43]. Engagement of the brain’s arousal and attention systems may mediate auditory–motor synchronization and facilitate action [44]. In this light, Leman [45] has suggested three (nested) levels of synchronized interaction. A first level is simple synchronization with the beat, which is considered a low-level interaction because beat induction is spontaneous and accessible [46]. A second level is embodied attunement, in which the dancers synchronize their movements with sound-energetic characteristics such as melody, harmony, tonality, or timbre, in addition with the beat. In this regard, Marinberg and Aviv [47] found that in choreographed solo dances, some professional dancers intrinsically tend to move in sync with the rhythm, whereas others move with the melodic phrases of the music. A third level is empathy, which relates to the synchronized bodily articulation with a musical expression–or temporal change of sound-energetic features, termed moving sonic forms–so that the bodily expression is united with the musical expression. The third level suggests a continuous narrative in dialogue with music, whereas the first level suggests a discrete micro-timing or clock. High aesthetic quality dance music performances are believed to have these levels closely nested, so SMS could provide timing cues for narrative empathic bodily articulations.

Overall, the literature suggests an influence of musical context on SMS, but the evidence based on micro-timing studies is very limited.

### Focus of the present study

The influence of musical context on dancers’ movements is studied here in the Promenade in Arabesque of the Odile variations. We focus on the Promenade in Arabesque as a prototypical example of a classical ballet dance technique in which the dancer jumps vertically on one foot in synchrony with the beats of the accompanying music and rotates the body in place while maintaining a certain pose in an arabesque (see the Supplementary Material section, or https://osf.io/ezn6w/files/osfstorage). Motion capture technology (MoCap) can accurately measure the timing of foot movements, while independent experts can indicate the timing of the beats in the music. Accordingly, the time of the movement onsets and the beats can be identified and the time interval between the movement onsets and the beats can be related to the time interval of two successive beats, giving the relative phase. Since we consider the time interval of two successive beats as one cycle (2 *π* in radians), we can express the sensiromotor (movement-beat) interval as a fraction of this cycle, also in radians.

**Fig 1:**
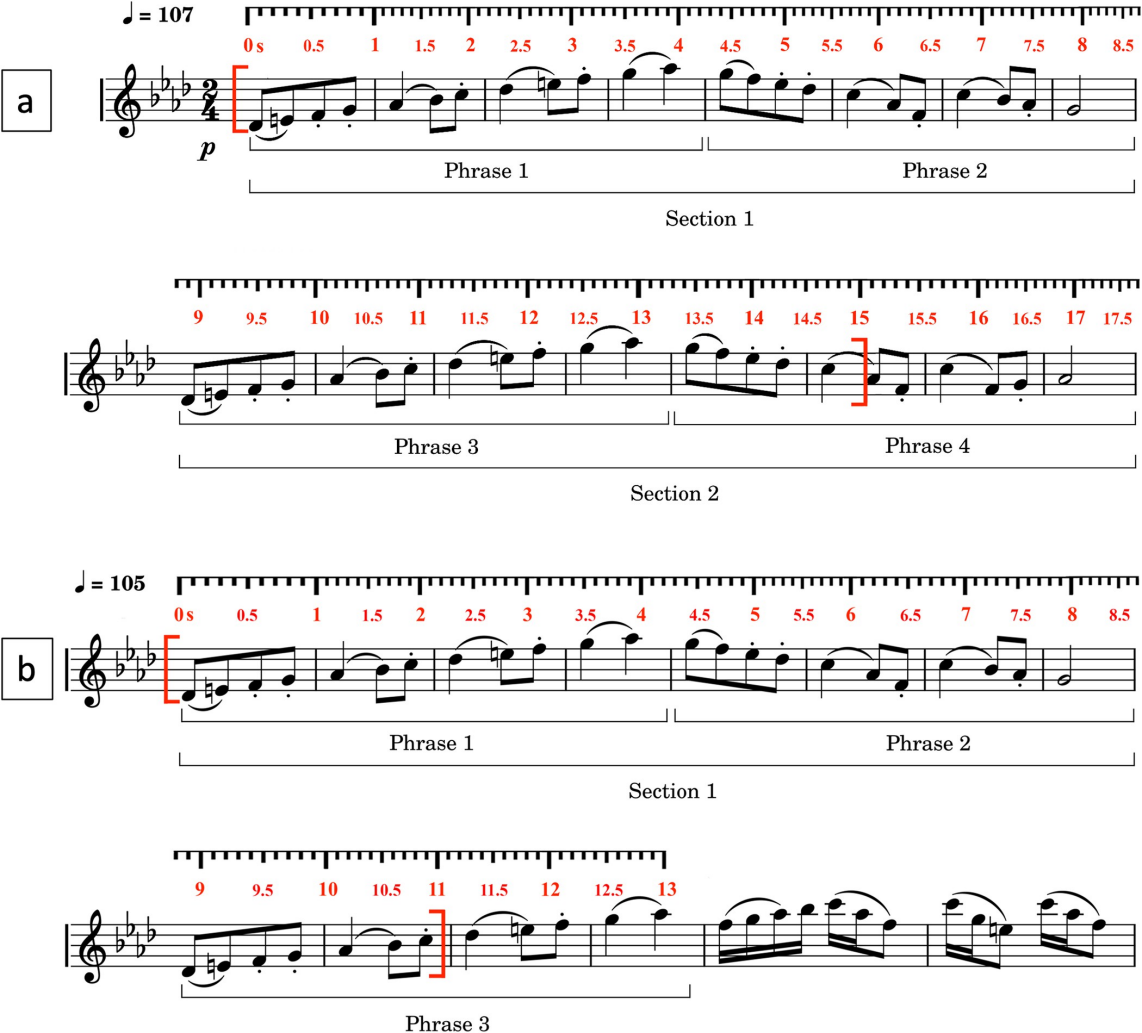
The score of the musical fragments from the Odile variation, act 3, Swan Lake. In a) I.F1 and F1 is displayed, consisting of two symmetric sections and four phrases. This fragment lasts 18 seconds, and the tempo is 107.0 (± 2.8) bpm for a 4^th^ note. In b) F2 is displayed, consisting of three phrases. This fragment lasts 13.7 seconds, and the tempo is 105.5 (± 2.2) bpm for a 4^th^ note. Fragments were truncated to 15 (I.F1 and F1) or 11 (F2) seconds to avoid artifacts resulting from the transition phase between dance techniques.

We investigate the influence of the musical context on the micro-timing of SMS by comparing three performances of the Promenade in Arabesque, henceforth called: dance-music fragments, or simple: fragments. The first fragment is isolated from (not embedded in) the surrounding musical context, whereas the second and third fragment are embedded in the surrounding musical context of the Odile variation (see Fig 1). Although we focus on the effect of musical context, that is, the repeats of patterns between and within the performed fragments, our contribution is also methodological in the sense that the analysis of SMS events, based on repeated measurements of a limited number of dancers, involves an analysis based on circular-linear smooth regression and circular statistics. Since SMS is time-correlated and probably adaptive to the occurrence of a repeat, or change in musical context during performance time, smooth regression allows us to capture dynamic changes of SMS during performance time, rather than just taking the mean over time (as is the case with circular statistics). The use of circular-linear smooth regression is by our knowledge new in this research domain. It is here introduced because it allows for the analysis of dynamic trends regarding the effect of musical context on the micro-timing of SMS, including some aspects of individual dancers’ expertise.

### Research hypotheses

We are interested to see whether musical context affects the dancer’s SMS to the beat. Musical context is understood in terms of repeats of patterns between and within music-dance fragments. The repeat between fragments is because fragments are similar (apart from the ending in Fragment 2). The repeat within fragments is due to repeats of phrases: phrase 1 is repeated in phrase 3, and phrase 2 is repeated in phrase 4, except for F2, where we don’t have a repeat of phrase 2, as shown in Fig 1.

The following hypotheses are investigated:

- Hypothesis 1: We expect the dancer’s operator foot to move vertically with the beat, in an in-phase beat synchronization. This can be measured as relative phase, by taking the ratio of the time interval between movement onset (Up Heel, Down Heel) and the preceding beat, and the time interval of the preceding beat and the successive beat. Accordingly, we can calculate the precision and the accuracy in the distribution of the relative phase, and we expect to see a negative mean asynchrony, meaning that the movement onsets before the beat, assuming the synchronization process is prediction based. With smooth regression, this NMA will be seen over performance time, offering a more detailed view on the dynamic of SMS over time.
- Hypothesis 2: We expect to see differences in the relative phase (reflected in either precision or accuracy) depending on the musical context in which the Promenade in Arabesque is performed. Between-fragments repeats can be analyzed by comparing distributions of the relative phase at time points corresponding to the repeats in the musical score. In particular, we expect that the non-embedded fragment may be different from the embedded fragment because of the difference in context. Within-fragment repeats can be analyzed in terms of dynamic means and variability of the relative phase using smooth regression. We expect that within each fragment, repeats show differences in anticipation values.
- Hypothesis 3: We do not expect a drift in SMS in repeated measures in such a short timescale, because the task involves skilled sensorimotor behavior. An SMS drift can be measured by checking differences in anticipation over the performance trials.

## Materials and methods

### Ethics

**Fig 2:**
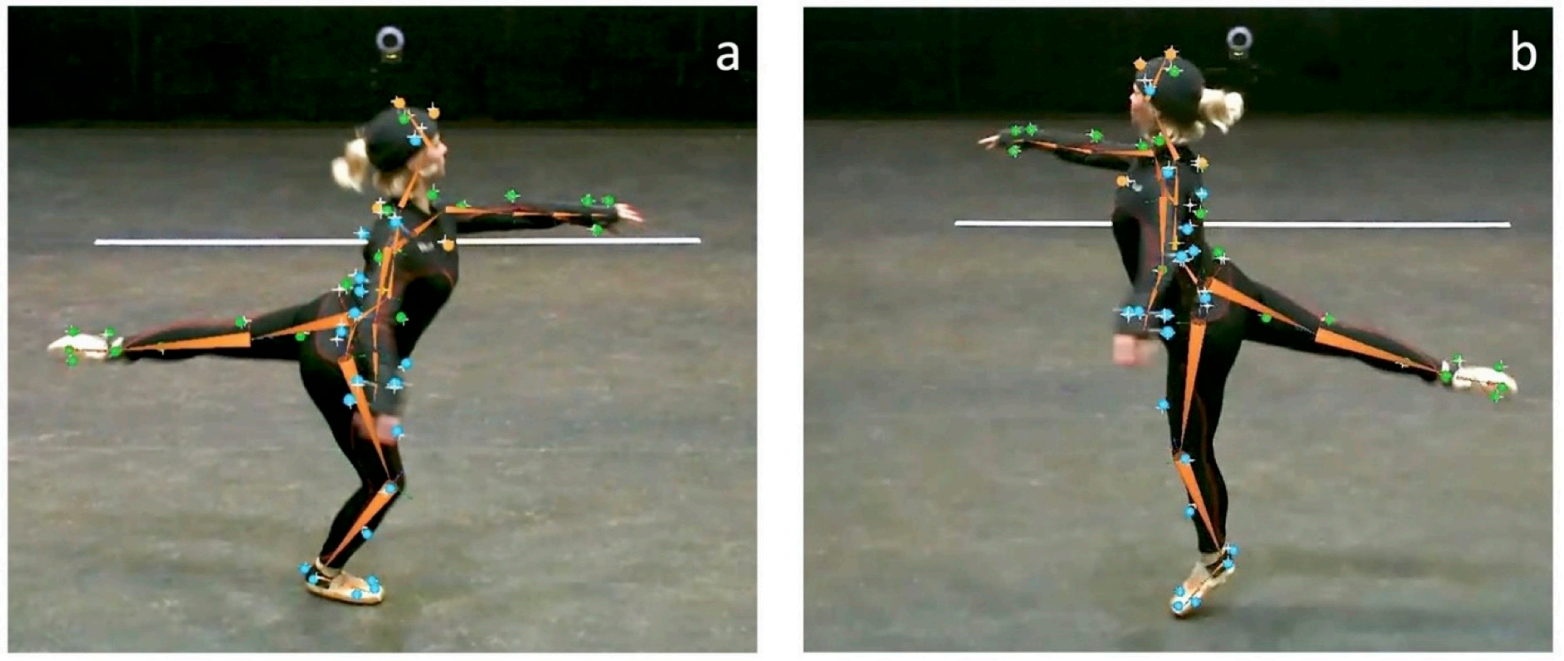
A still of one of the participants performing the Promenade in Arabesque figure. The participant hops on one foot, lifting the heel periodically while slowly pivoting around her axis. a) Display of the Heel Down movement onset (when the participant’s heel touches the floor). b) Display of the Heel Up movement onset (when the participant’s heel is at its maximal vertical position). Blue and green dots indicate the marker positions.

The research was approved by the Research Ethics Committee (REC) of the Faculty of Science, Radboud University Nijmegen, the Netherlands (REC19041). The individual in Fig 2 has given written informed consent (as outlined in the PLOS consent form) to publish these case details.

### Participants

Participants needed to be active female ballet dancers, either professional or amateur, and they needed to be familiar with the Odile variations. Four female classical ballet dancers participated in the study (P1, P2, P3, P4), two of whom (P1 and P2) were non-professional dancers (14 and 18 years) with eight and six years of classical ballet training, respectively. Two dancers (P3 and P4) were professionals (19 and 35 years old) with six years of classical ballet training and one with 17 years of professional performance background. The participants had little musical training, except for P3, who had regular musical training and played musical instruments for seven years. In addition to these four dancers, we have data of one amateur participant (P0). The recording took place in the Art and Science Interaction Laboratory (ASIL) of the Institute for Psychoacoustic and Electronic Music (IPEM) in the Department of Art History, Musicology, and Theater Sciences of Ghent University, Belgium.

### Music stimulus

The music stimulus was the Odile variation, act 3 from Swan Lake ballet, composed by Tchaikovsky. The original choreography was developed by Julius Reisinger in 1877 and then revised and re-created by Marius Petipa and Lev Ivanov in 1895 [48]. This variation was selected because it is generally believed to be one of the masterpieces of female classical ballet variations. Choreographers typically use the Promenade in Arabesque figure in this variation to embody the Odile character (e.g., Olga Smirnova’s performance from seconds 10 to 23 in [49]). This piece of music has a clear rhythmic structure, and the composition can be seen as consisting of 13 sections, which can be referred to as (A, B, C, D, D, E, E, D, D’, F, F, G, H). The total time is 83 seconds, and the time signature is 2/4. Typically, the Promenade in Arabesque figure is performed in section D and D’.

Dancers were requested to perform an isolated fragment consisting of sections 4 and 5 (or D, D) (hereafter referred to as fragment I.F1), and, dancers were requested to perform the entire variation, which contains sections 4 and 5 (or D, D) (hereafter referred to as fragment F1) and sections 8 and 9 (or D, D’; hereafter referred to as fragment F2). Foot movements become irregular after 15 in F1, and after 11 seconds in F2, due to the transition to a different dance technique. Therefore, fragment 2 was truncated to the first 11 seconds of the dance (Fig 1), whereas the fragment I.F1 and F1 were truncated to the first 15 seconds. Besides the between-fragments repeats, each fragment has a within-fragment repeat of a musical phrase, starting at bar 9.

In summary, our analysis focuses on the performance of the Promenade in Arabesque, in three different musical contexts. Apart from a performance of I.F1, which has no surrounding musical context, the same dance figure is performed in a surrounding musical context, during F1 and its reiteration F2. There are between-fragment repeats and within-fragment repeats of the same musical material, and only F2 has a slightly different pattern at the end, due to a musical climax.

### Procedure

**Fig 3:**
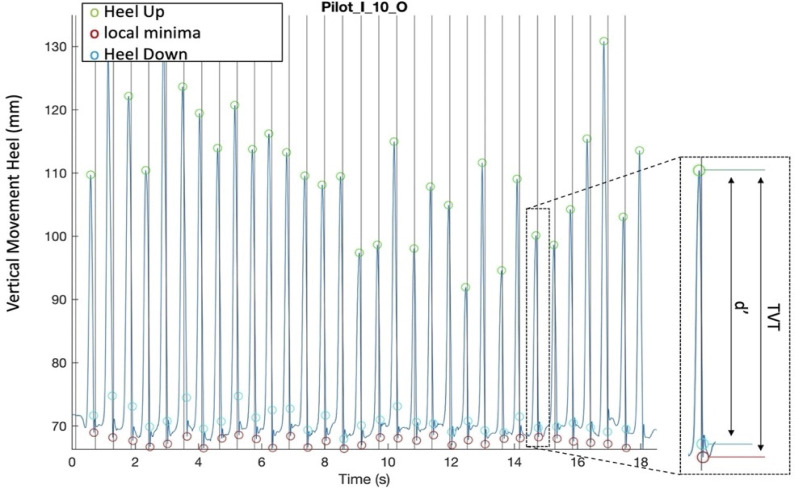
The vertical movement of the left heel during a dance fragment. The local maxima (green circles) are onset data (Heel Up). The local minima (red circles) show a little bounce, so the first time point where the marker is recorded at >90% (D’) of its total vertical trajectory (TVT) between a local maximum and its subsequent minimum (light blue circles) is considered as the second set of onsets (Heel Down). The black vertical lines indicate the musical beats during the dance fragment.

The sessions started by informing the participants about the objectives of the study and the expected tasks. The participants were then asked to complete the required informed consent form. Participants were free to stop the experiment at any time. All participants were familiar with the music stimuli and the choreography in advance. They were asked to wear a special MoCap suit, with 42 reflective markers per the instructions of the Qualisys Animation Marker Set (see Fig 2). This marker set allows quantitative analysis of foot movements (see Fig 3). To ensure an accurate movement recording, the participants were requested to move within a 64 m^2^ (8m by 8m) square to remain within view of the MoCap cameras. The participants’ movements were captured with 18 infrared MoCap cameras running Qualisys Track Manager (QTM, Qualisys, Göteborg, Sweden) software, at a frame rate of 120 Hz (fps). In addition, audio and video performances were recorded for our reference by four RGB cameras placed at each corner. The musical stimuli were played through speakers, which were installed as standard in the laboratory. The sound volume was adjusted before recording in accordance with the participants’ preference. MoCap, video, and audio data streams were synchronized after recording using an SMTPE clock.

### Tasks

The performances were recorded in two stages. Initially, participants were asked to perform fragment I.F1, performing this 12 times one after another (so we obtained 12 measurements of I.F1, referred to hereafter as trials 1–12). All participants were free to perform the technique with the foot with which they are most dexterous, but they were asked to use the same foot throughout the experiment when performing the technique. A musical stimulus was always counted in. The participants were given ample time to rest between trials to minimize fatigue. The first stage lasted about 90 minutes.

During the second stage, participants were asked to dance along the entire variation (consisting of both fragments F1 and F2). Again, they had to do this 12 times in a row. However, in our study design, we manipulated the musical signal of repeat 5 and 8 to see the effect of phase change and period change. The results of these manipulations are not reported in the present study, and the data are therefore omitted. In the end, we obtained 10 measurements of both F1 and F2. When performing the Promenade in Arabesque throughout the variation, participants were asked to use the same foot as in the first stage (a video sample of each of the four participants dancing the I.F1, F1, and F2 fragments is available in the Supplementary Material section, or at https://osf.io/ezn6w/files/osfstorage). However, throughout the rest of the variation, the participants were free to dance as they pleased. The second stage also lasted about 90 minutes. The recording sessions and a short break were followed by a face-to-face interview with each participant who self-assessed their performance (not reported here). All in all, the experiment lasted about 3 hours and 40 minutes per participant.

### Data processing

After recording, all markers were identified semi-automatically using the QTM software. Then, each MoCap dataset was synchronized with the audio and video recordings and merged into one dataset [50].

### Detection of beats

The beats (here defined as the time at which a pulse marks a regular tempo) were determined manually for all fragments. To specify the beats, a musically trained researcher tapped along with the music on a key of a computer keyboard while the respective music fragments were playing. Each time a key was pressed down, a timestamp was recorded, and this procedure was repeated 10 times per fragment. The final beats (per fragment) were determined by taking the average of the 10 timestamps of each measured beats.

### Detection of movement onsets

**Fig 4:**
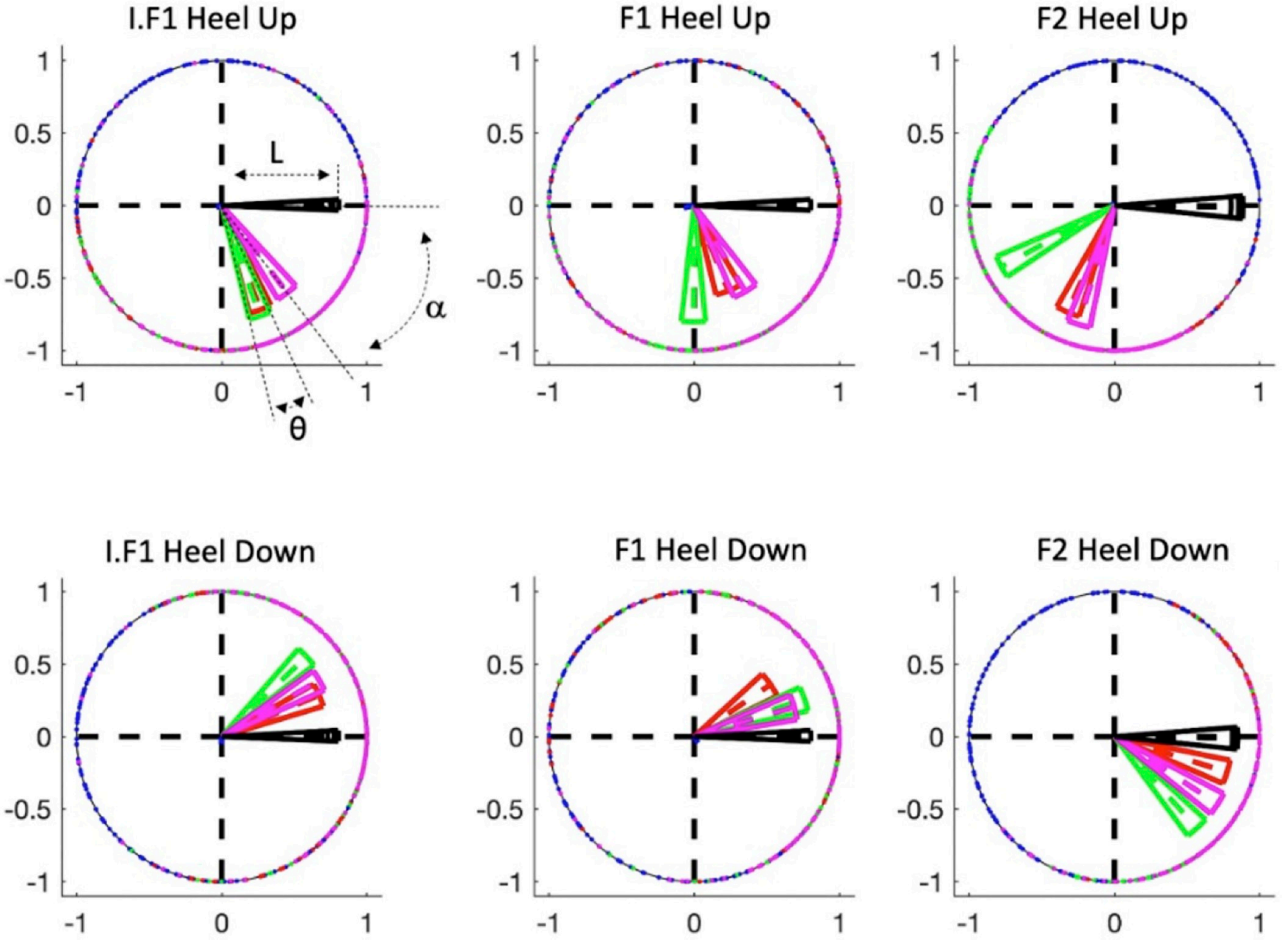
Circular statistics representation. Colored vectors represent, for each participant (P1 to P4 as blue, red, green, and magenta sectors, respectively), the angular direction (α) of a specific onset relative to the beat (black vector), as well as the magnitude of this direction (L). Vectors (for clarity, represented as the dotted line in the center of the wedge) are shown for each fragment (I.F1, F1, and F2), and the central angle θ of the vectors represents the 95% confidence interval of its angular direction. P1’s vector is so small that it is not visible in this figure. Confidence intervals are also given for the beat onsets.

Having analyzed the videos of the dance recordings, we observed that the main rhythmical component of the feet movement was determined by the up and down movement of the left or right heel (depending on the participant’s preference) relative to the floor. From the MoCap data, therefore, only the vertical position coordinates of the heel marker data stream were used for further analysis (Fig 3). For each dance fragment, the local maxima and minima of the vertical heel movement were detected. The local maxima were one set of movement onsets (hereafter referred to as Heel Up, see Figs 3 and 4). The local minima had some minor artifacts because of some bouncing movement of the marker after the heel hit the floor. Hence, the other set of movement onsets was determined as the first sampled time point where the marker was at >90% of its total vertical trajectory between a local maximum and its subsequent minimum (hereafter referred to as Heel Down, see Figs 3 and 4).

### Synchronization of movement and beat onsets

The rationale behind using circular statistics is that we can examine SMS data from a circular distribution, allowing a comparison of SMS between the 3 different fragments. The circular statistical analysis complements the smooth regression analysis. The movement onset data (Heel Up and Heel Down) was projected onto an angular scale based on the beat onset data:

$${ha}_{i}=2*\pi *\frac{\left(h_{i}-b_{j}\right)}{\left(b_{j+1}-b_{j}\right)}$$ (1)

Where h_i_ applies to each of the N movement onset data points (with i = 1, 2, …, N) occurring between every pair of consecutive beats (b_j_, b_j+1_), (with j = 1, 2, …, M-1) if there are M beats in total. The ha_i_ is the projection of h_i_ to the angular scale and 0 ≤ ha_i_ ≤ 2 π (Fig 4).

Subsequently, the mean vector direction (α) and the mean vector length (L) were calculated per participant per measurement using the Circular Statistics Toolbox [51] for each measurement (see Fig 4):

$$r_{i}=\left[\begin{array}{c} cos(ha_{i})\\ sin({ha}_{i}) \end{array}\right]$$ (2)

$$\bar{r}=\sum_{i=1}^{N}r_{i}$$ (3)

Here, i = 1, 2, …, N are the N movement onset points as described earlier. α is the angle of $\bar{r}$, as retrieved by the four-quadrant inverse tangent function, and L is the norm of $\bar{r}$.

α describes the offset between the movement onset and the beat onset, or the accuracy of the synchronization. When α is negative, the movement onset anticipates the beat onset. When α is positive, it lags behind the beat onset. L describes the consistency of the timing of the movement onset relative to the beat onset, or the precision of the synchronization. 0 ≤ L ≤ 1 and the higher L, the better the precision.

Per fragment, per participant, it was tested whether the movement onset data displayed a specific angular direction relative to the beat using the Hodges-Ajne test [51, 52], followed by a Watson-Williams multi-sample test to determine whether the direction was significantly different from the beat (Table 1) [51, 52]. In this and subsequent analysis using circular statistics, all fragments were truncated to the first 11 seconds to avoid possible differences due to differences in execution time and context. In particular, F2 differs from I.F1 and F1 because of a climax effect that we avoided in our analysis (see Fig 1).

**Table 1:** Results of circular statistics analysis.

Movement onset	Dancer	P_dir_			P_sync_			α (rad)			L		
		I.F1	F1	F2	I.F1	F1	F2	I.F1	F1	F2	I.F1	F1	F2
**Heel Up**	1	0.67	0.38	0.92	/	/	/	/	/	/	0.040	0.074	0.033
	2	<< 0.01	<< 0.01	<< 0.01	<< 0.01	<< 0.01	<< 0.01	-1.22 ± 0.10	-1.19 ± 0.14	-1.98 ± 0.10	0.765 ± 0.051	0.633 ± 0.076	0.802 ± 0.058
	3	<< 0.01	<< 0.01	<< 0.01	<< 0.01	<< 0.01	<< 0.01	-1.239 ± 0.094	-1.58 ± 0.10	-2.645 ± 0.089	0.806 ± 0.044	0.801 ± 0.0061	0.886 ± 0.055
	4	<< 0.01	<< 0.01	<< 0.01	<< 0.01	<< 0.01	<< 0.01	-0.93 ± 0.10	-1.03 ± 0.13	-1.863 ± 0.094	0.752 ± 0.074	0.689 ± 0.084	0.856 ± 0.040
**Heel Down**	1	0.53	0.62	0.96	/	/	/	/	/	/	0.052	0.051	0.029
	2	<< 0.01	<< 0.01	<< 0.01	<< 0.01	<< 0.01	<< 0.01	0.40 ± 0.11	0.60 ± 0.14	-0.31 ± 0.10	0.730 ± 0.062	0.64 ± 0.10	0.813 ± 0.046
	3	<< 0.01	<< 0.01	<< 0.01	<< 0.01	<< 0.01	<< 0.01	0.756 ± 0.097	0.33 ± 0.11	-0.836 ± 0.098	0.804 ± 0.072	0.802 ± 0.051	0.849 ± 0.072
	4	<< 0.01	<< 0.01	<< 0.01	<< 0.01	<< 0.01	<< 0.01	0.525 ± 0.099	0.29 ± 0.12	-0.579 ± 0.097	0.777 ± 0.064	0.718 ± 0.053	0.849 ± 0.066

P_sync_ << 0.01 indicates that the data have a mean direction (α), i.e., that there is a consistent phase relationship, or synchrony, using the Hodges-Ajne test. P_phase_ << 0.01 indicates that the data are not in phase with the beats using the Watson-Williams multi-sample test. α is the vector angle (± circular standard deviation), and L is the vector length (± standard deviation). I.F1 is isolated fragment 1, F1 is fragment 1, F2 is fragment 2.

### Regression modelling

Circular-linear (hierarchical) regression is used to predict the relative phase values, using a von-Mises link function to map the predictions to the response interval [-π to + π]. The population-level predictors are Fragment (I.F1, F1, F2) and Heel (Up or Down). In subsequent models Participant (2 to 4, considering 1 as an outlier) is either handled as population-level predictor or group-level predictor. Trial (the 10 or 12 different trials of each fragment, each trial representing a measurement) is always handled as group-level predictor. The circular-linear (hierarchical) models that include Time use an expansion of the time-varying data in a 10-dimensional space using thin-plate splines and penalized regression, thus accounting for time-correlated data and overfitting in the form of smoothing [53]. In all analyses, fragment 2 was truncated to 11 seconds, while the other fragments were truncated to 15 seconds. The models are implemented with the R package brms [54] running on a 48-dual-core server, allowing for parallel processing (see also Supplementary Materials for details).

To test hypothesis 1, whether there are differences in and between fragments, we calculate the posterior predictive distributions. For each distribution we then estimate the mean, and its uncertainty in terms of 95% of the probability mass in a critical interval from lower to upper. Next, we calculate the direction of probability mass below zero and above zero of the difference of two distributions, obtaining a measure of the strength of evidence in favor of the hypothesis of contrast. We focus here on contrasts between Fragment, Heel, over all participants and repeated measures (i.e., all performances of P2, P3, P4 taken together). An interesting outcome is the standard deviation of the group-level predictor Trial, as it reflects the impact of repeated measures. Accordingly, the circular-linear (hierarchical) regression model is specified in brms syntax as:

Model H1H3:

$$bf\left(\begin{array}{c} Phase∼1+Heel*Fragment*Participant+(1|Trial),\\ kappa∼1+Heel*Fragment*Participant+(1|Trial) \end{array}\right)$$ (4)

The test hypothesis 3, whether there is an effect of repeated measures, is based on this model H1H3. We draw samples from the posterior distribution of the group-level Trial, and we evaluate the obtained distributions using the direction of probability as measure.

To test hypothesis 2, whether relative phase depends on musical context, we use a circular-linear hierarchical smooth regression model. In that model, we no longer assume that the response is a distribution of time-independent taps (of Heel Up or Heel Down). Instead, we consider that taps are the result of a non-linear system with time-dependency in outputs due to brain predictions and effector control depending on musical context. Accordingly, we estimate a nonlinear effect over time (generalizing across trials and participants) using smooth terms. In brms syntax, this regression model is specified as:

Model H2a:

$$bf\left(\begin{array}{c} Phase∼1+interaction(Heel,Fragment)\\ +s(Time,by=interaction(Heel,Fragment)\\ +(1|Trial+Participant),\\ kappa∼1+interaction(Heel,Fragment)\\ +s(Time,by=interaction(Heel,Fragment)\\ +(1|Trial+Participant) \end{array}\right)$$ (5)

Our outcome of interest is time-dependent trends in Phase, related to repeats between Fragments, and repeats within Fragments, separating Heel Up and Heel Down. Using draws from posterior predictions, defined over time segments, we calculate contrasts using the direction of probability as measure. For illustrative purposes, we run a second model with a focus on individual participants, including now also P0. This model estimates the smooths for each participant. In brms syntax, this model is specified as:

Model H2b:

$$bf\left(\begin{array}{c} Phase∼1+interaction(Participant,Heel,Fragment)\\ +s(Time,by=interaction(Participant,Heel,Fragment)\\ +(1|Trial),\\ kappa∼1+interaction(Participant,Heel,Fragment)\\ +s(Time,by=interaction(Participant,Heel,Fragment)\\ +(1|Trial) \end{array}\right)$$ (6)

The outcome of interest is time-dependent trends in Phase and differences between Participants and Fragments, focusing on Heel Down.

## Results

### Circular statistics

The probability that there is a direction (P_sync_) << 0.01 acquired by the Hodges-Ajne test shows that the data have a mean direction (α), i.e., that there is a consistent phase relationship, or synchrony. The probability that the movement onsets are perfectly in phase with the beat (P_phase_) << 0.01 acquired by the Watson-Williams multi-sample test shows that the data are not in phase with the beats (Table 1).

To compare the calculated α and L in the different fragments, a non-parametric multiple comparison test was applied for each participant separately (Table 2).

**Table 2:** Results of a non-parametric multiple comparisons test.

			Heel Up (p-value)		Heel Down (p-value)	
	Dancer	Condition	I.F1	F2	I.F1	F2
**L**	1	F1	0.77	0.61	0.99	0.82
		F2	0.21		0.88	
	2	F1	**0.043**	0.086	0.060	0.072
		F2	0.98		1.0	
	3	F1	0.96	**0.022**	0.99	0.16
		F2	**0.035**		0.010	
	4	F1	0.52	0.75	0.39	0.94
		F2	0.16		0.61	
**α**	1	F1	0.70	0.53	0.98	0.78
		F2	0.13		0.65	
	2	F1	0.99	**<0.01**	0.62	**<0.001**
		F2	**<0.01**		**<0.01**	
	3	F1	0.25	**<0.01**	0.15	**0.013**
		F2	**<<0.001**		**<<0.001**	
	4	F1	0.81	**0.019**	0.68	**0.018**
		F2	**<0.01**		**<0.001**	

p is the probability that α or L is equal across different fragments (I.F1, F1, and F2) for each participant separately.

To detect the metrical level on which the onsets interact with the beats, the ratio of the inter-onset interval (IOI) with the inter-beat interval (IBI) was calculated (IOI:IBI). An error margin of this metric was calculated based on the standard deviations of both IOI and IBI. If the ratio is, e.g., 0.5, this means that there are two movement onsets per beat on average, or, e.g., if the ratio is 3, this means that there are three beats per movement onset. The ratios are calculated per participant, per fragment (Table 3).

**Table 3:** Ratios of inter-onset intervals with the inter-beat interval.

	Heel Up			Heel Down		
Dancer	I.F1	F1	F2	I.F1	F1	F2
**1**	0.837 ± 0.064	0.800 ± 0.059	0.771 ± 0.058	0.832 ± 0.037	0.801 ± 0.041	0.776 ± 0.037
**2**	0.992 ± 0.033	0.985 ± 0.033	0.981 ± 0.032	0.992 ± 0.027	0.987 ± 0.026	0.988 ± 0.027
**3**	0.997 ± 0.035	0.995 ± 0.037	0.988 ± 0.037	0.995 ± 0.034	0.990 ± 0.033	0.985 ± 0.034
**4**	1.001 ± 0.039	0.999 ± 0.041	0.991 ± 0.037	0.996 ± 0.029	0.991 ± 0.028	0.989 ± 0.028

The ratios of the inter-onset interval (IOI) of both Heel Up and Heel Down with the inter-beat interval (IBI), or IOI: IBI (± error), per participant, per fragment (I.F1, F1, and F2).

Because of the small sample size, no statistical tests were performed to determine the differences between professional and non-professional participants at group level.

The feet of P2, P3, and P4 move in synchrony with the musical beats in all three fragments, for both movement onset data Heel Up and Heel Down, as reflected in the high L values (L > 0.62, see Table 1). In contrast, P1 does not dance synchronously with the music in any of the fragments. There is no difference in synchrony between fragments (see Table 2).

Synchrony can be further explored by looking more closely at the ratio of the inter-onset interval (IOI) and the inter-beat interval (IBI) (see Table 3). For P2, P3, and P4, the ratio IOI:IBI is approximately equal to 1, indicating that both Heel Up and Heel Down interact with the music at the tatum level or the 4^th^ notes. Consequently, P1’s, IOI:IBI is remarkably close to 0.8, suggesting that she is timing approximately in quintuplets of 4^th^ notes, so five movement onsets evenly spread of over every group of four beat onsets.

The latter observation means that we do not find a consistent phase relationship between the movement onsets and the beat onsets (or the angular direction α), and that there is no synchronicity (or L is very small). It must be noted that this result depends on the metric level chosen for our analysis (see section 2.9), as we investigate the phase relationship of the movement onsets relative to every 4^th^ note.

The movement onsets of P2, P3, and P4 are not perfectly in phase with the beat in any of the fragments (see Table 1). The movement onsets are slightly different between F1 and F2, and between I.F1 and F2 in most of the cases, for P2, P3, and P4 (see Tables 1 and 2). Considering the Heel Down movement onsets, this means that participants are lagging behind the beat in F1 and I.F1 and anticipating the beat in F2. Heel Up is in general timed about a ¼ of a beat interval before Heel Down.

The movement onsets of P2, P3, and P4 are in synchrony with the beat onsets in all three fragments, as reflected in the L value (L > 0.62, see Table 1). P1, in contrast, has a timing of the onsets approximately in quintuplets of 4^th^ notes, so five movement onsets evenly spread over each group of four beat onsets, and we omitted this participant from our further analyses.

**Fig 5:**
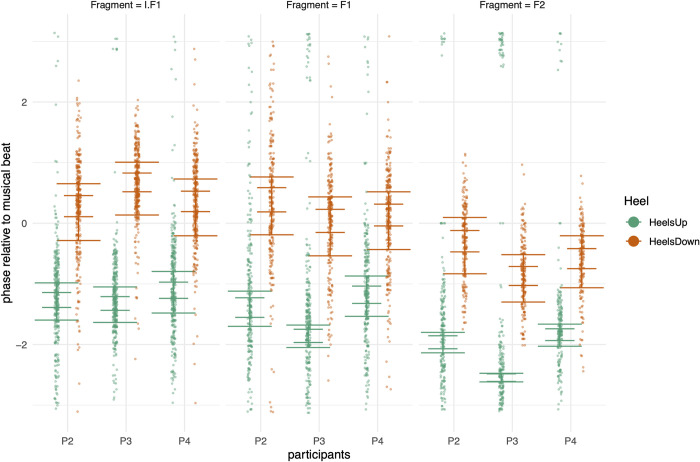
Results of model H1H3. Short horizontal error bars indicate the CI with 95% probability mass in favor of the expected value of the posterior predictive distribution, excluding the group-level effect of Trial. The long horizon error bars include the group-level effect of Trial.

Although the Heel Down onset is synchronized with the beat onset of the 4^th^ notes (-0.84<α<0.76), the onsets of P2, P3, and P4 are not perfectly synchronized in phase with the beat in any of the fragments (see Table 1, Figs 4 and 5). Despite the high L value, they are lagging behind the beat in F1 and I.F1, whereas they are anticipating the beat in F2. In contrast, Heel Up is arbitrarily timed (-2.65<α<-0.93) at about ¼ of a beat before Heel Down, not coinciding with a particular rhythmic subdivision but rather depending on the dancer’s individual preference. It is remarkable that, despite its arbitrary timing relative to the meter, the accuracy of the Heel Up onsets is even slightly better than the accuracy the Heel Down onsets (see Table 1, Fig 4).

### Regression modelling

A more detailed understanding of the relative phase distribution is obtained with regression modelling. A posterior prediction check of the fitted models reveals a close resemblance between the distribution of replicated data and the distribution of original data, indicating that the models are well fitted [55].

Contrasts based on model H1H3 can be checked in Fig 5 and in Table 4. Fig 5 shows the expected value of the posterior prediction distributions and their critical intervals at 95%. Each bar depicts (a) the uncertainty without the effect of repeated measure variance (inner horizontal bars) and (b) the uncertainty with the effect of repeated measure variance (outer horizontal bars). Overall, Fig 5 suggests that fragment F2 is distinct from fragment I.F1 and fragment F1. Generally, the critical intervals are wider for Heel Down compared with Heel Up. Fragment F2 has a narrow critical interval for Heel Up, indicating that the mean remains stable, over repeated measures. Fragment F2 stands out as distinct from fragment I.F1 and fragment F1.

**Table 4:** Contrasts of fragments.

row	fragment	heel	pd>0	estdiff	lowdiff	uppdiff	est1	est2
1	I.F1, F1	Heel Down, Heel Down	100	0.22	0.11	0.31	0.48	0.17
2	F1, F2	Heel Down, Heel Down	100	0.77	0.65	0.87	0.17	-0.59
3	I.F1, F2	Heel Down, Heel Down	100	0.90	0.81	1.00	0.48	-0.59
4	I.F1, F1	Heel Up, Heel Up	100	0.33	0.24	0.43	-1.29	-1.64
5	F1, F2	Heel Up, Heel Up	100	0.65	0.55	0.76	-1.64	-2.26
6	I.F1, F2	Heel Up, Heel Up	100	0.95	0.86	1.03	-1.29	-2.26

Calculated contrasts are shown in Table 4. The table reveals that all contrasts, although different in size (see the column estdiff) are different.

The column “fragment” contains Fragment pairs whose contrast is calculated. Column "heel" indicates the Heel level. Column “pd>0” shows the direction of probability (in %) above zero, with above 95% indicating strong evidence for contrast. Columns “estdiff lowdiff uppdiff” show the mean and the lower and upper CI-values of the calculated difference between the posterior predictive distribution of the first fragment, and the posterior predictive distribution of the second fragment. The columns “est1” and “est2” show the estimated mean of these distributions.

The group-level effect of Trial has a standard deviation of about 0.13, indicating that the effect is small. When considering the individual effects of Trial (considering each repeated measure or Trial separately) a trend can be discerned, showing higher contribution with higher repeated measure, suggesting that the lag of the movement onsets relative to the beat onsets increases slightly over repeated measures, showing a drift in SMS.

**Fig 6:**
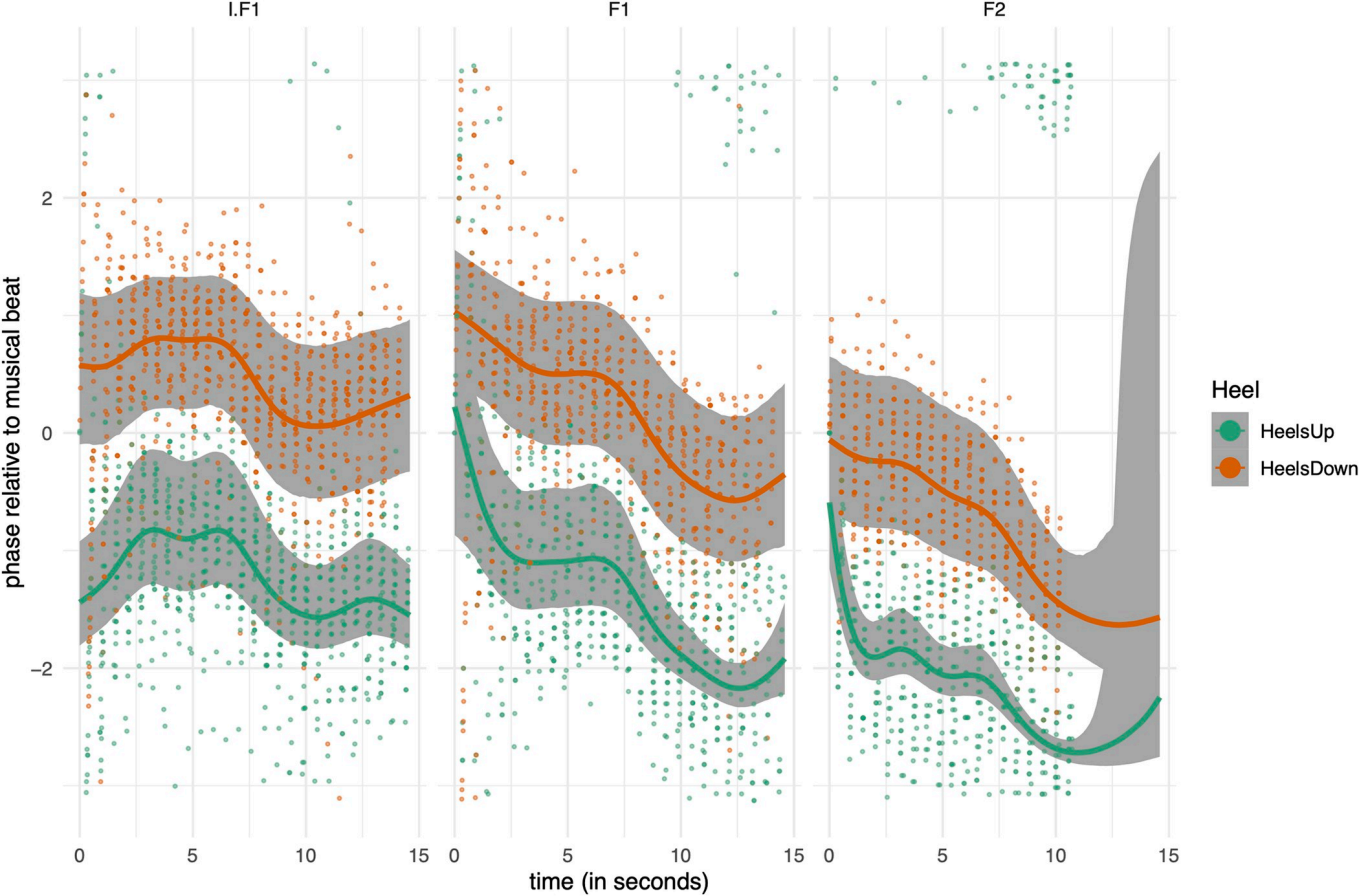
Results of model H2a. Dynamic means (full lines) and 95% critical intervals (bands) of posterior predictions overlayed with the raw data (small dots). Horizontal axis is performance time, vertical axis is phase (in radians).

**Fig 7:**
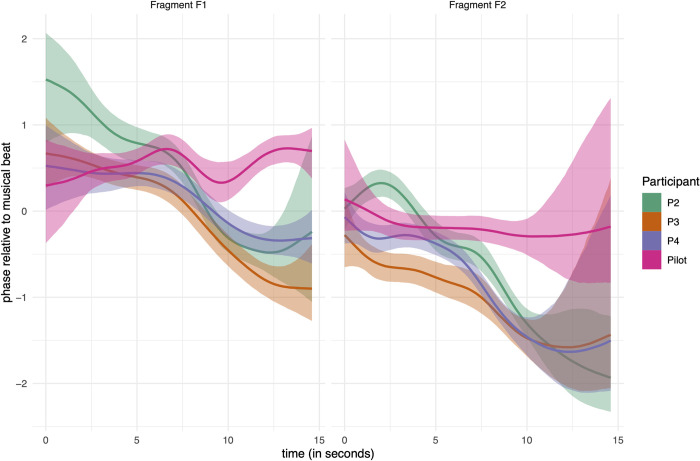
Results of model H3b. Dynamical means (full lines) and 95% critical intervals (bands) of posterior predictions of Heel Down. Horizontal axis is performance time, vertical axis is phase (in radians).

Fig 6 offers a view on SMS during performance time, generalized over repeated measures and participants. Overall, the trend is that synchronization becomes more anticipatory as time proceeds. Fig 7 shows the smooths per participant over performance time of Heel Down. In this figure, the pilot of dancer P0 is included. For analysis purposes, the first 2 seconds can be discarded because the variability is considerable due to inconsistency in the movements of the dancers in the beginning of each fragment in combination with smoothing artifacts. The uncertainty of the dynamical mean (shown in full lines) is shown in the bands, indicating 95% of the probability mass, as defined by the lower and upper border of each band. The bands offer a visual indication of whether sections can be considered significantly different from each other.

Within fragments it is possible to observe regions where the relative phase values are stable over time, as well as regions where the relative phase values change. The data of fragment I.F1 show that relative phase values are at a plateau of about slightly above -1 radians for Heels Up, and this plateau emerges at about 2 seconds and lasts up to about 6.5 seconds. This plateau is then followed by a decrease in relative phase values to about -1.5 radians, resulting in a new plateau from second 9 onwards. This new plateau occurs in accord with section 2 in the score, which is an exact repeat of section 1 (see Fig 1). A similar pattern emerges in F1. In fragment F2 a plateau of relative phase values in the vicinity of -2 radians can be observed, lasting up to about 7 seconds, followed by a decrease.

Contrast calculations between segments are given in Table 5 (see also Supplementary Materials). Based on the time annotated music score (Fig 1), we consider the following contrast types:

- Between-repeats 1–3: this is a contrast of a segment from 0 to 4.25 seconds of phrase 1 and a segment 2 from 9 to 13.25 seconds of phrase 3.
- Between-repeats 2–4: this is a contrast of a segment from 4.26 to 6 seconds of phrase 2 and a segment 2 from 13.26 to 15 seconds of phrase 4.
- Within-section 1: this is a contrast of a segment from 2.51 to 4.25 seconds of phrase 1 and a segment from 4.26 to 6 seconds of phrase 2.
- Within-section 2: this is a contrast of a segment from 11.51 to 13.25 seconds of phrase 3 and a segment from 13.26 to 15 seconds of phrase 4.
- Between-fragments: this contrast omits the first 2 seconds of the fragment, avoiding large variability in data due to the beginning of the dance.

**Table 5:** Contrasts of segments.

row	contrast	fragment	heel	pd>0	estdiff	lowdiff	uppdiff
1	C1 between-repeats 1–3	I.F1, I.F1	Heel Down, Heel Down	100.00	0.54	0.39	0.66
2	C2 between-repeats 2–4	I.F1, I.F1	Heel Down, Heel Down	100.00	0.57	0.48	0.69
3	C3 within-section 1	I.F1, I.F1	Heel Down, Heel Down	10.48	-0.05	-0.13	0.03
4	C4 within-section 2	I.F1, I.F1	Heel Down, Heel Down	1.12	-0.11	-0.19	-0.01
5	C1 between-repeats 1–3	F1, F1	Heel Down, Heel Down	100.00	1.04	0.86	1.21
6	C2 between-repeats 2–4	F1, F1	Heel Down, Heel Down	100.00	1.01	0.85	1.17
7	C3 within-section 1	F1, F1	Heel Down, Heel Down	98.84	0.14	0.02	0.24
8	C4 within-section 2	F1, F1	Heel Down, Heel Down	46.36	0.00	-0.12	0.11
9	C1 between-repeats 1–3	F2, F2	Heel Down, Heel Down	100.00	1.04	0.94	1.15
10	C3 within-section 1	F2, F2	Heel Down, Heel Down	100.00	0.19	0.09	0.29
11	Contrast between-fragments	F1, F2	Heel Down, Heel Down	100.00	0.81	0.75	0.87
12	C1 between-repeats 1–3	I.F1, I.F1	Heel Up, Heel Up	100.00	0.42	0.30	0.54
13	C2 between-repeats 2–4	I.F1, I.F1	Heel Up, Heel Up	100.00	0.58	0.47	0.68
14	C3 within-section 1	I.F1, I.F1	Heel Up, Heel Up	52.28	0.01	-0.10	0.09
15	C4 within-section 2	I.F1, I.F1	Heel Up, Heel Up	30.08	-0.03	-0.10	0.07
16	C1 between-repeats 1–3	F1, F1	Heel Up, Heel Up	100.00	1.20	0.95	1.39
17	C2 between-repeats 2–4	F1, F1	Heel Up, Heel Up	100.00	1.00	0.84	1.16
18	C3 within-section 1	F1, F1	Heel Up, Heel Up	85.76	0.06	-0.05	0.18
19	C4 within-section 2	F1, F1	Heel Up, Heel Up	48.88	-0.01	-0.12	0.11
20	C1 between-repeats 1–3	F2, F2	Heel Up, Heel Up	100.00	0.98	0.86	1.08
21	C3 within-section 1	F2, F2	Heel Up, Heel Up	99.40	0.17	0.03	0.31
22	Contrast between-fragments	F1, F2	Heel Up, Heel Up	100.00	0.74	0.68	0.81

The column “contrast” indicates the contrast type. For the meaning of other columns, see Table 4.

Phrases are defined in the annotated score (Fig 1). More specifically, phrases 1 and 2 define section 1, and phrases 2 and 4 define section 2, except for F2 which lacks phrase 4. Between-repeats contrasts compare segments having similar musical content. Within-section contrast compare two segments within a phrase having different musical content. The segments are defined such that relevant sections are cut out, selecting equal time intervals if possible. Repeated phrases are called repeats.

Table 5 reveals strong evidence for a difference among between-repeats contrasts. In all these contrasts, the estimated value (estdiff) is around 0.5 rad in I.F1, and 1 rad in F1 and F2, meaning that the segment 1 has a higher relative phase value than segment 2. Otherwise stated: segment 2 is anticipated more than segment 1. The within-section contrasts tell a different story showing a small, estimated value (estdiff), sometimes in negative direction, suggesting an effect that can be neglected. Finally, the contrast between F1 and F2 is confirmed, when the first 2 seconds are discarded.

Overall, it can be concluded that within fragments, a repeated musical content is more anticipated than the first occurrence of that musical content. Phrases having different musical content tend to be equal in terms of anticipation.

Finally, the results of model H2b (Fig 7) show SMS over time from the viewpoint of individual participants, generalized over repeated measures. Fig 7 shows only the Heel Down data for fragment F1 and F2. It can be observed that P2 (novice dancer) and P0 (occasional dancer, less developed than the novice) behave differently compared to P3 and P4 (professional dancers). In particular, P2 is less stable in the first four seconds, whereas P0 is rather sensitive to the between-fragments (F1, F2) repeats than to the within-fragment repeats.

## Discussion

In this study, we investigated SMS in classical ballet solo dance, using a methodology to analyze the relative phase of heel movement and musical beat. Circular statistics, and circular-linear (smooth) regression modelling were used to investigate SMS during the performance and over different repetitions of the same performance. In general, the results show an anticipation effect related to musical context.

According to the circular statistics analysis, the movement onsets of participants 2, 3 and 4 are in synchrony with the beat onsets in all 3 fragments, as reflected in the L value (L > 0.62, see Table 1) and in the 95% critical intervals (Fig 5). Participant 1, in contrast, has a timing of the onsets approximately in quintuplets of 4th notes, so 5 movement onsets evenly spread over every group of 4 beat onsets, and we omitted this participant from our further analyses.

While the Heel Down onset is synchronized with the beat onset of the 4th notes (-0.84<α<0.76), the onsets of participants 2, 3, and 4 are in none of the fragments perfectly synchronized in phase with the beat (see Table 1, Figs 4 and 5). Despite the high L value, they are lagging the beat in F1 and I.F1, while they are anticipating the beat in F2. In contrast, Heel Up is arbitrarily timed (-2.65<α<-0.93) at about ¼ of a beat before Heel Down, not coinciding with a particular rhythmic subdivision but rather depending on the individual preference of the dancer. It is remarkable that, despite its arbitrary timing relative to the meter, the accuracy of the Heel Up onsets is even slightly better than the accuracy the Heel Down onsets (see Table 1, Figs 4 and 5).

The consistency of out-of-phase onsets is also reported in other studies [56]. Remarkably, the placement in time of the Heel Up onsets relative to Heel Down is only slightly different for every participant, and independent of the context (i.e., the lag between Heel Up and Heel Down, is specific for every participant, but it stays constant in the different fragments F1, F2 and I.F1). The inter-onset interval between Heel Up and Heel Down remains constant, but it does not coincide with a particular rhythmical entity, suggesting that the dancers choose an arbitrary interval to time this onset and stick to it during their performance. The timing of this interval could be rather dependent on factors like anatomy, tempo, and practice, while being less influenced by artistic or expressive factors [56]. The observation that precision of both tasks is similar, while the underlying mechanisms might be different, is interesting and requires more research.

The phase relationship between movement and beat onsets differs between fragments, while vector length (or critical interval), basically, is not different between fragments. Heel Down movements are lagging in I.F1 and F1, but they are anticipating in F2. However, the lag is less than the reaction time [1]. It can therefore be stated that, in I.F1 and F1, participants are still predicting the beat, while the movement onset is timed a bit after the beat.

Circular statistics is suited to analyze differences between fragments I.F1, F1 and F2. However, a circular-linear (hierarchical and smooth) regression modelling approach allows for a time resolved analysis, highlighting dynamic changes in SMS during performance, as well as differences in SMS between between-fragments and within-fragment repetitions.

Contrasts of entire fragments reveal that the expected value of the posterior prediction distributions of fragment I.F1, F1 and F2 differ from each other in a direction showing increasing anticipation. In addition, a non-significant, positive trend in the delay between the onset of the beat and the onset of movement due to repeated measures (Trial) can be observed. The observed trend may be due to factors such as fatigue [57]. Since we find no significant evolution of the SMS across trials (neither towards anticipation nor towards delay), we hypothesize that the synchronization is based on (automated) sensory-motor coupling rather than a cognitively mediated coupling.

Contrasts of segments in fragments reveal that the expected value of the posterior prediction distribution of repeats have a higher contrast than non-repeats, suggesting an effect of repeats in the direction of more anticipation of about 0.5 up to 1 radian, which amounts to 45 up to 90 milliseconds (0.56 * 0.5 / 2pi = 0.045 s).

Analysis with a focus on individual dancers confirms an anticipation effect due to repeat and musical context in both P3 and P4 (the experts). In contrast, we observed some instability in SMS of P2 at the beginning of the fragments, but this was followed by the anticipation effect eventually. In contrast, P0 shows only a weak anticipation effect. Whether these differences between beginner and amateur versus professional are a group characteristic remains to be investigated in future studies with more participants.

Our findings can be related to prediction processing theory, in particular the idea that SMS builds up prediction schemes allowing anticipation [1]. The difference in intercept between F1 and F2 suggest that a prediction scheme established in F1 is used and reinforced for the timing of the repeated fragment, thus allowing more anticipation in F2. This interpretation is supported by the fact that the starting relative phase value of fragment F2 is close to the ending relative phase value of fragment F1, thus showing more anticipation. However, in between F1 and F2 there is a musical context that possibly influenced the timing of F2. Hence, the effect might not be related to the repeat of F1 as such, but to the repeat of F1 and its subsequent musical content.

Much stronger evidence for a direct effect of musical context on SMS is found in the within-fragment repeats, suggesting an influence of previous musical context during the repeat in terms of more anticipation, in a range that is in line with NMA ranges in the literature [1, 18]. In short, the within-fragment repeats provide evidence in favor of the idea that musical context influences sensorimotor anticipation. This observation can be interpreted in line with a prediction processing theory that accounts for prediction memory, meaning that a previously established prediction scheme gests reinforced during a repeat of the musical content in which it was first established.

Our data show more anticipation at the end of fragment F1 and even a more pronounced anticipation slope at the end of fragment F2, suggesting possible other influences on SMS. Although we have less direct evidence available, we don’t exclude that the observed anticipation is influenced by emotional engagement in addition to the predictive model being established. In music, the effect is known as a "forward timing" effect, and it is generally considered as an important component of expression. The effect can also be attributed to emotional arousal generated by the expected climax in the musical development. Fragment F2 has a clear build-up to a climax (see the score in Fig 1), and the anticipation of the musical beat could probably be related to that climax, similar to the so-called "playing forward" in jazz [58]. By timing the movements ahead of time, the dancer can evoke a sense of energy and excitement, thus enhancing the dance expression. Accordingly, the anticipated movements at the end of F2 could be an expression of that phenomenon, in addition to the influence of prediction schemes.

We believe that the circular-linear (hierarchical and smooth) regression, including the possibility of contrasting segments in and between smooths, offers a workable tool for further research about the effect of musical context on SMS. The dynamic in the micro-timing of SMS and its breakdown to relevant segments in line with repeated patterns in musical context, points to an interesting memory effect on prediction that needs further investigation.

### Limitations

Several limitations of the present study may be mentioned. The beat annotation was done manually, and it is likely that the annotated beat onsets precede the actual beats to some degree [2]. This means that the NMA of the dancers is likely to be underestimated or even masked when comparing movement onsets with the annotated beat onsets, possibly masking NMA in F1 and I.F1. Additionally, given that beat onsets were measured by a human operator, the annotated beat onsets approximate the actual beat onsets with a certain accuracy and precision. To evaluate the accuracy of the annotated beat onsets, the actual music was overlaid with a beep sound triggered by the annotated onsets. Despite eventual NMA in the beat annotation, this was not noticeable when performing the latter test. Therefore, we decided to consider the annotated beat onsets as ground truth, calibrating the circular statistics on these values. The repeated annotations were used to calculate the precision of the annotated onsets. This precision was used to perform statistics. Moreover, it is known that SMS can differ according to the body part performing the movement [8]. Moreover, with increased distance from the ears to the heels, and increased inertia, as compared to e.g., finger tapping, it is possible that predictive behavior can result in lagging movement onsets reported in F1 and I.F1 [59].

Several technical challenges arose during this work, concerning the precise detection of beat and movement onsets. Music and dance were carefully chosen to achieve a reliable paradigm for SMS detection in dance, while investigating its relation to the musical and narrative context. However, the paradigm used in this study, was optimized to study dance-music interactions that are repetitive, on a clear beat. To study more complex movements, or involving music lacking a clear beat, the paradigm will likely need some adjustments. Moreover, several factors are believed to affect the ecological validity of this study. The participants were restricted to a 64 m2 area for MoCap, the laboratory floor was not suited for dancing with pointe shoes, and the participants had to wear a MoCap suit with optical markers, including markers on their pointe shoes, possible hindering their movements. Finally, the duration of the experiment, exhausted the participants, eventually causing physical discomfort near the end of the experiment.

The calculation of all (Bayesian) regression models, given a calculation server with 48 dual cores, took several hours to generate 8000 iterations of the mcmc algorithm on a STAN engine. The computational cost may pose a challenge for future large-scale datasets, especially when more dancers are involved.

Finally, due to the limited number of participants, it is not feasible to generalize our results, especially in view of group effects such as novices versus professionals. However, we were able to perform repeated measures that form a reliable set for claims about drift in individual dancers. Accordingly, our methodology is adapted to this focused approach.

## Conclusion

In conclusion, in line with Hypothesis 1 the dancers synchronize (spontaneously) in phase to the beat. Circular statistics and smooth regression revealed relative phase differences between and within fragments. In line with Hypothesis 2, our data suggest that the relative phase values change as the musical context in which the Promenade in Arabesque is performed varies. Repetitions then mark a corresponding increase in anticipation. In line with Hypothesis 3 no drift of SMS towards more pronounced NMA or lag could be observed, rather we observed increase in variance possibly related to fatigue.

The present study contributes to a better understanding of micro-timing in SMS in classical ballet. Overall, it seems that the SMS, recruiting prediction schemes for micro-timing, can be influenced by context factors, in particular repeats in musical context. This finding is relevant for better understanding of prediction processes in humans, and it may lead to quantifications of micro-timing sensitivity in time-critical domains of application such as in music, sports, and possibly sensorimotor-based physiotherapy.

It is likely that our findings extend to other forms of dancing, but further research would be needed to confirm this conjecture. Further research is also needed to handle group-level effects, between gender, between solo dance (variation), couple dance (Pas de deux), and group dances (Corps de ballet), and to study the aesthetic effect on audiences.

## Future prospects

The hypothesis that emotional arousal may influence SMS is supported by music practice, where rhythm and SMS are known to be important components of expression, both in melodic instruments (e.g., through rubato playing of Chopin’s music) as in rhythmic instruments (e.g., forward, laid-back or “in the pocket” playing in a jazz rhythm section) [60]. In dance performance, however, this expressive timing is far less well understood [61] and more research is needed to investigate this relationship.

The assumption of contextual influence on micro-timing is supported by the conjecture that prediction schemes supporting SMS are influenced by factors of musical context relevant to prediction, such as repetitions. The repetition is a potentially predictive factor influencing micro-timing because it is based on the same pattern that was previously observed and acted upon, making it likely to leave a prediction-related trace for micro-timing in the memory. Accordingly, a plausible hypothesis is that a repeat could recruit the previously established micro-timing memory trace and use it to reinforce current micro-timing predictions. Thus, a repetition can affect SMS micro-timing by providing an already established micro-timing prediction scheme, leading to more anticipation. More anticipation can be seen as an expression of prediction. Thus, musical context, particularly repetitions, can be hypothesized to interfere with SMS prediction schemes, making the micro-timing more anticipatory. This phenomenon ties in with the idea of embodied attunement [45], which states that moving along with music is influenced by contextual facts, or with the idea that cognitive processing influences sensorimotor processing.

## Supporting information

S1 Data The data as fed into ModelH1H3. (RDATA)

S2 Data The data as fed into ModelH2a. (RDATA)

S3 Data The data as fed into ModelH2b. (RDATA)

S1 Protocol A detailed protocol of the regression modelling analysis. (PDF)

S2 Protocol A depiction of the workflow of data acquisition and analysis. (PDF)

S1 Movie Dancer 1 dancing the complete variation. (MKV)

S2 Movie Dancer 1 dancing the isolated fragment. (MKV)

S3 Movie Dancer 2 dancing the complete variation. (MKV)

S4 Movie Dancer 2 dancing the isolated fragment. (MKV)

S5 Movie Dancer 3 dancing the complete variation. (MKV)

S6 Movie Dancer 3 dancing the isolated fragment. (MKV)

S7 Movie Dancer 4 dancing the complete variation. (MKV)

S8 Movie Dancer 4 dancing the isolated fragment. (MKV)
