# Supplementary material for: Influence of musical context on sensorimotor synchronization in classical ballet solo dance
Source: PLoS One. 2023 Apr 18;18(4):e0284387. doi: 10.1371/journal.pone.0284387 (PMC10112816; doi:10.1371/journal.pone.0284387)
Supplement: S1 Protocol — (PDF) [file pone.0284387.s004.pdf]

Supplementary Material

2022-11-13

This document shows analysis results of the circular-linear (smooth) regression modelling related to “Influence of musical context on sensorimotor synchronization in classical ballet solo dance”

The musical context

Both scores below are annotated with ruler on top, representing time in seconds. The figure below shows the score for I.F1 and F1, with 15 seconds of used data.

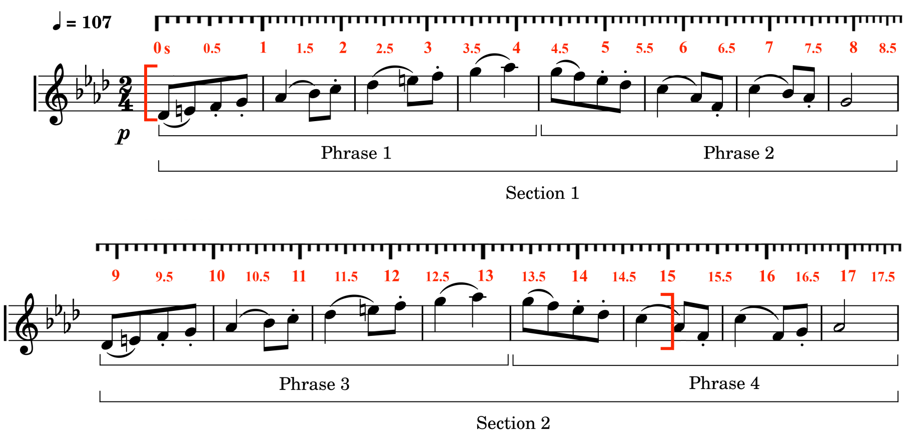

The figure below shows the score for F2, with 11 seconds of used data.

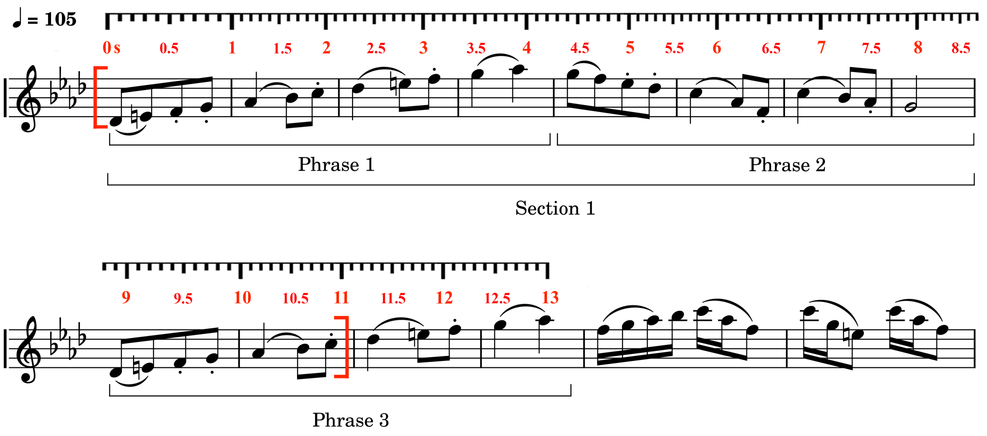

A quick view on the data

This is the basic dataset for different models:

- time = absolute time of the Heel onset in the piece
- time0 = time relative to the beginning of the fragment
- phase = as calculated using 0 to 2*pi
- Phase1 = rotated to -pi to pi
- Phase 2 = other rotation not used
- Trialf = trial as factor
- Trial = trial as integer
- Participant = subject taking part in the experiment, P2, P3, P4, or Pilot
- Fragment = dance-music fragment, either I.F1, F1, F2
- Heel = either HeelsDown or HeelsUp

```
##       phase    Phase1    Phase2 Trialf Trial Participant Fragment      Heel
## 1 2.9943425 2.9943425 -2.430435     01     1          P2       F1 HeelsDown
## 2 2.1839685 2.1839685  3.042376     01     1          P2       F1 HeelsDown
## 3 1.7258352 1.7258352  2.584243     01     1          P2       F1 HeelsDown
## 4 1.3545494 1.3545494  2.212957     01     1          P2       F1 HeelsDown
## 5 0.4342826 0.4342826  1.292690     01     1          P2       F1 HeelsDown
## 6 6.1170850 -0.1661003  0.692307     01     1          P2       F1 HeelsDown
##       time0
## 1 0.2720699
## 2 0.7640699
## 3 1.2800699
## 4 1.8050699
## 5 2.2800699
## 6 2.7890699
```

```
##       phase           Phase1            Phase2            Trialf
##  Min.   :0.000   Min.   :-3.1261   Min.   :-3.14058   03     : 417
##  1st Qu.:1.157   1st Qu.:-1.5120   1st Qu.:-0.74575   10     : 417
##  Median :4.515   Median :-0.6925   Median : 0.05417   01     : 415
##  Mean   :3.672   Mean   :-0.6052   Mean   : 0.07778   02     : 415
##  3rd Qu.:5.339   3rd Qu.: 0.2611   3rd Qu.: 1.01324   06     : 415
##  Max.   :6.279   Max.   : 3.1391   Max.   : 3.13282   07     : 415
##                                                       (Other):1948
##       Trial        Participant Fragment        Heel           time0
##  Min.   : 1.000   P2:1444     I.F1:1826   HeelsUp  :2269   Min.   : 0.000
##  1st Qu.: 3.000   P3:1486     F1  :1490   HeelsDown:2173   1st Qu.: 3.325
##  Median : 7.000   P4:1512     F2  :1126                    Median : 6.590
##  Mean   : 6.498                                            Mean   : 6.606
##  3rd Qu.:10.000                                            3rd Qu.: 9.617
##  Max.   :12.000                                            Max.   :14.592
##
```

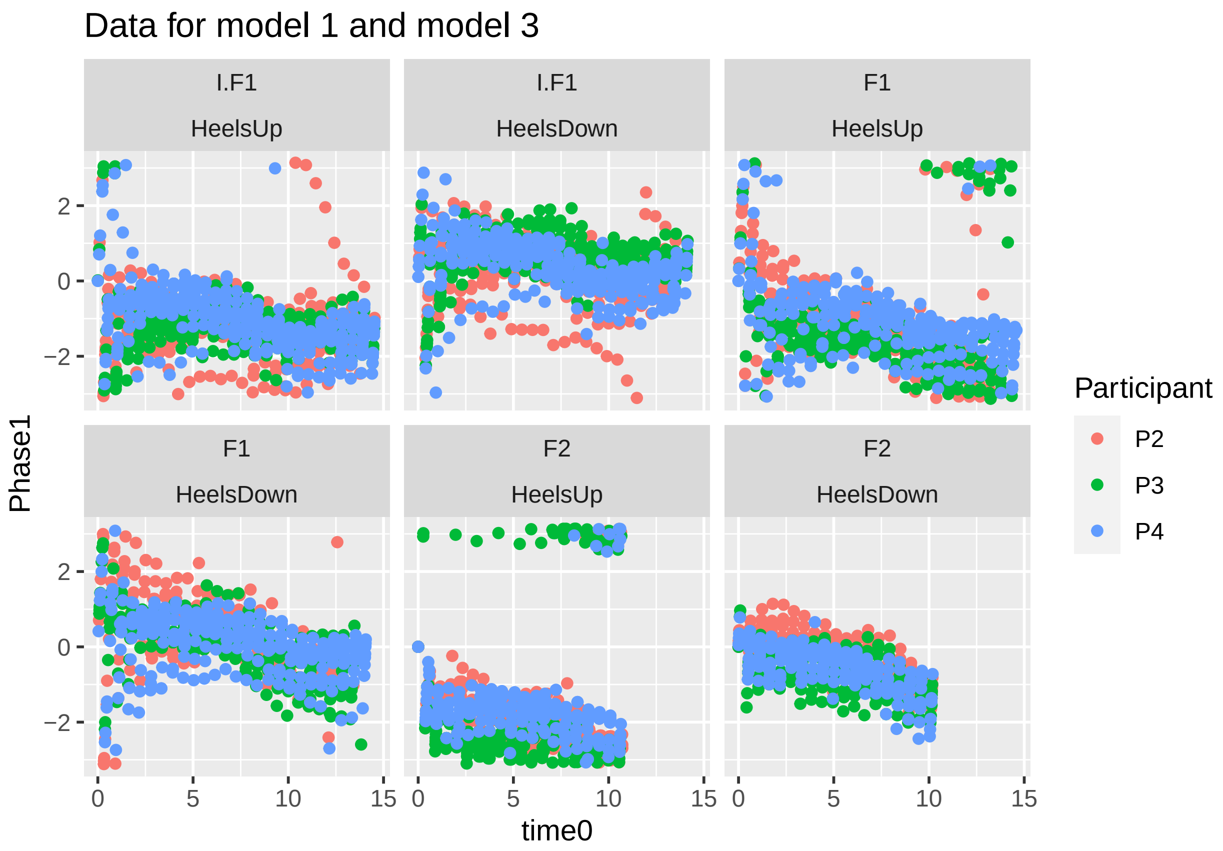

Statistical approach for testing

To test hypothesis 1, whether there are differences in and between fragments, we calculate posterior predictive distributions, using the `posterior_epred` function in R-package `brms`. This function computes the expected value of the posterior predictive distribution. The rationale is that we summarize over subjects, looking for effects of a mean defined by repeated measures in a small population. Alternatively, the `posterior_prediction` function would compute the posterior predictive distribution including resid-

ual variance. However, that option is more suited for counterfactual predictions. See the discussion at https://github.com/paul-buerkner/brms/issues/1408.

For each distribution we then estimate the mean and its uncertainty in terms of 95% of the probability mass in a critical interval from lower to upper. Next we calculate the direction of probability mass below zero and above zero, of the difference of two distributions, obtaining a measure of the strength of evidence in favour of the hypothesis of contrast, rather than the hypothesis of no contrast. We focus here on contrasts between `Fragment`, `HeelsDown` and `HeelsUp`, over all participants and repeated measures (i.e. all performances of P2, P3, P4 taken together).

To check hypothesis 2, whether there is an effect of repeated measures, we draw samples from the posterior distribution of the group-level `Trialf` and we evaluate the obtained values.

To test hypothesis 3, whether there are differences in between-repeats and non-repeats within-segments, we define two segments of time and calculate for these segments the posterior predictions and contrasts as described above.

Hypothesis 1: differences between fragments

H1: Diagnostics

The diagnostics of model 1 shows the posterio predictive check and the main variables.

```
## Phase1 ~ 1 + Fragment * Participant * Heel + (1 | Trialf)
## kappa ~ 1 + Fragment * Participant * Heel + (1 | Trialf)
```

```
## Loading required package: Rcpp
```

```
## Loading 'brms' package (version 2.18.0). Useful instructions
## can be found by typing help('brms'). A more detailed introduction
## to the package is available through vignette('brms_overview').
```

```
##
## Attaching package: 'brms'
```

```
## The following object is masked from 'package:stats':
##
##     ar
```

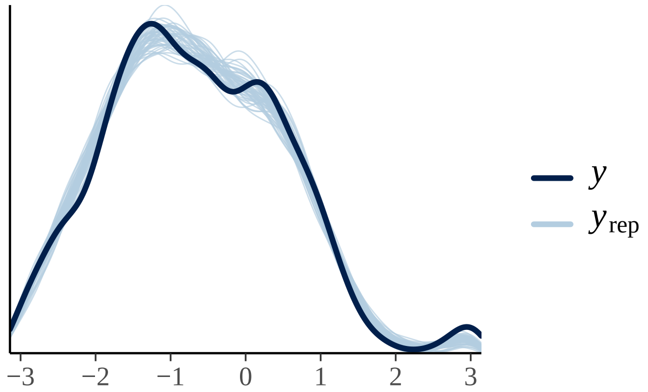

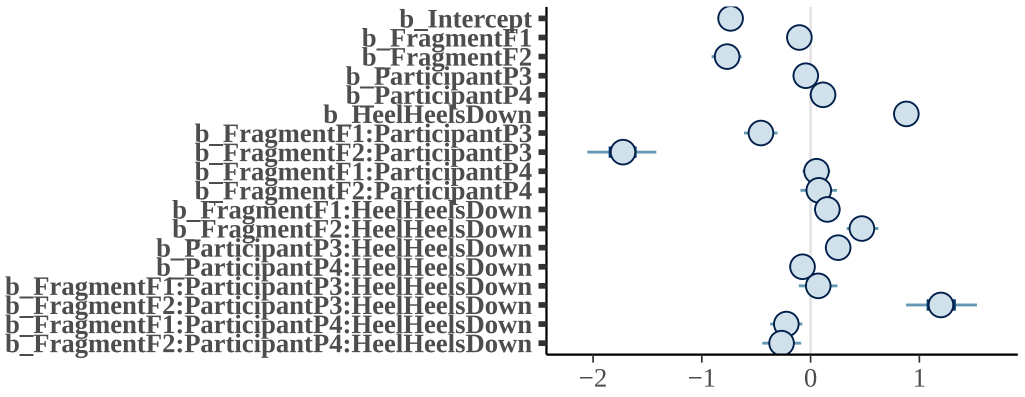


```
##  Family: von_mises
##   Links: mu = tan_half; kappa = log
## Formula: Phase1 ~ 1 + Fragment * Participant * Heel + (1 | Trialf)
##          kappa ~ 1 + Fragment * Participant * Heel + (1 | Trialf)
##    Data: Data (Number of observations: 4442)
##   Draws: 2 chains, each with iter = 8000; warmup = 3000; thin = 2;
##          total post-warmup draws = 5000
##
## Group-Level Effects:
## ~Trialf (Number of levels: 12)
##                      Estimate Est.Error l-95% CI u-95% CI Rhat Bulk_ESS Tail_ESS
## sd(Intercept)            0.13      0.03     0.08     0.22 1.00     2853     3856
## sd(kappa_Intercept)      0.21      0.06     0.12     0.36 1.00     2595     3658
##
## Population-Level Effects:
##                                          Estimate Est.Error l-95% CI
## Intercept                                   -0.74      0.05    -0.83
## kappa_Intercept                              1.04      0.10     0.85
## FragmentF1                                  -0.10      0.06    -0.23
## FragmentF2                                  -0.77      0.08    -0.94
## ParticipantP3                               -0.04      0.04    -0.12
## ParticipantP4                                0.12      0.04     0.04
## HeelHeelsDown                                0.88      0.03     0.81
## FragmentF1:ParticipantP3                    -0.46      0.09    -0.65
## FragmentF2:ParticipantP3                    -1.73      0.19    -2.12
## FragmentF1:ParticipantP4                     0.05      0.08    -0.10
```

```
## FragmentF2:ParticipantP4                          0.07      0.10    -0.12
## FragmentF1:HeelHeelsDown                          0.16      0.07     0.01
## FragmentF2:HeelHeelsDown                          0.47      0.09     0.30
## ParticipantP3:HeelHeelsDown                       0.25      0.05     0.16
## ParticipantP4:HeelHeelsDown                      -0.08      0.05    -0.17
## FragmentF1:ParticipantP3:HeelHeelsDown            0.07      0.11    -0.14
## FragmentF2:ParticipantP3:HeelHeelsDown            1.20      0.20     0.83
## FragmentF1:ParticipantP4:HeelHeelsDown           -0.22      0.09    -0.40
## FragmentF2:ParticipantP4:HeelHeelsDown           -0.27      0.11    -0.48
## kappa_FragmentF1                                 -0.59      0.12    -0.82
## kappa_FragmentF2                                 -0.09      0.12    -0.31
## kappa_ParticipantP3                               0.29      0.10     0.09
## kappa_ParticipantP4                              -0.06      0.10    -0.26
## kappa_HeelHeelsDown                              -0.02      0.10    -0.22
## kappa_FragmentF1:ParticipantP3                    0.07      0.16    -0.24
## kappa_FragmentF2:ParticipantP3                    0.20      0.17    -0.13
## kappa_FragmentF1:ParticipantP4                    0.47      0.16     0.17
## kappa_FragmentF2:ParticipantP4                    0.52      0.16     0.21
## kappa_FragmentF1:HeelHeelsDown                    0.06      0.16    -0.25
## kappa_FragmentF2:HeelHeelsDown                    0.23      0.16    -0.09
## kappa_ParticipantP3:HeelHeelsDown                -0.01      0.14    -0.30
## kappa_ParticipantP4:HeelHeelsDown                 0.15      0.14    -0.13
## kappa_FragmentF1:ParticipantP3:HeelHeelsDown     -0.11      0.22    -0.54
## kappa_FragmentF2:ParticipantP3:HeelHeelsDown     -0.46      0.23    -0.90
## kappa_FragmentF1:ParticipantP4:HeelHeelsDown     -0.07      0.22    -0.51
## kappa_FragmentF2:ParticipantP4:HeelHeelsDown     -0.34      0.23    -0.80
##                                              u-95% CI Rhat Bulk_ESS Tail_ESS
## Intercept                                        -0.64 1.00     3360     4092
## kappa_Intercept                                   1.23 1.00     3645     4208
## FragmentF1                                        0.02 1.00     3898     4151
## FragmentF2                                       -0.61 1.00     4073     4039
## ParticipantP3                                     0.03 1.00     4109     4243
## ParticipantP4                                     0.19 1.00     4285     4331
## HeelHeelsDown                                     0.95 1.00     4067     4629
## FragmentF1:ParticipantP3                         -0.27 1.00     4418     4399
## FragmentF2:ParticipantP3                         -1.37 1.00     4133     4299
## FragmentF1:ParticipantP4                          0.20 1.00     3951     4079
## FragmentF2:ParticipantP4                          0.27 1.00     4178     4440
## FragmentF1:HeelHeelsDown                          0.30 1.00     3840     3822
## FragmentF2:HeelHeelsDown                          0.65 1.00     3956     4123
## ParticipantP3:HeelHeelsDown                       0.34 1.00     3955     3654
## ParticipantP4:HeelHeelsDown                       0.02 1.00     3563     4306
## FragmentF1:ParticipantP3:HeelHeelsDown            0.28 1.00     4192     4450
## FragmentF2:ParticipantP3:HeelHeelsDown            1.60 1.00     4041     3833
## FragmentF1:ParticipantP4:HeelHeelsDown           -0.05 1.00     3713     4059
## FragmentF2:ParticipantP4:HeelHeelsDown           -0.05 1.00     4005     4505
## kappa_FragmentF1                                 -0.37 1.00     3997     4170
## kappa_FragmentF2                                  0.14 1.00     3953     4151
## kappa_ParticipantP3                               0.49 1.00     3969     4047
## kappa_ParticipantP4                               0.14 1.00     3868     4460
## kappa_HeelHeelsDown                               0.18 1.00     3856     4122
## kappa_FragmentF1:ParticipantP3                    0.38 1.00     3850     4426
## kappa_FragmentF2:ParticipantP3                    0.53 1.00     4133     4265
## kappa_FragmentF1:ParticipantP4                    0.78 1.00     3943     4255
```

```
## kappa_FragmentF2:ParticipantP4                    0.83 1.00     3788     4279
## kappa_FragmentF1:HeelHeelsDown                     0.39 1.00     3913     4164
## kappa_FragmentF2:HeelHeelsDown                     0.54 1.00     3939     4303
## kappa_ParticipantP3:HeelHeelsDown                  0.26 1.00     3906     3958
## kappa_ParticipantP4:HeelHeelsDown                  0.42 1.00     3937     4122
## kappa_FragmentF1:ParticipantP3:HeelHeelsDown       0.34 1.00     3811     3813
## kappa_FragmentF2:ParticipantP3:HeelHeelsDown      -0.01 1.00     3962     4512
## kappa_FragmentF1:ParticipantP4:HeelHeelsDown       0.36 1.00     3984     4358
## kappa_FragmentF2:ParticipantP4:HeelHeelsDown       0.10 1.00     3912     4401
##
## Draws were sampled using sample(hmc). For each parameter, Bulk_ESS
## and Tail_ESS are effective sample size measures, and Rhat is the potential
## scale reduction factor on split chains (at convergence, Rhat = 1).
```

H1: Posterior predictions (retrodiction)

Short horizontal error bars indicate the CI with 95% probablity mass in favour of the expected value of the posterior predictive distribution, excluding the group-effect of `Trial`. The long horizon error bars include the group-effect of `Trial`.

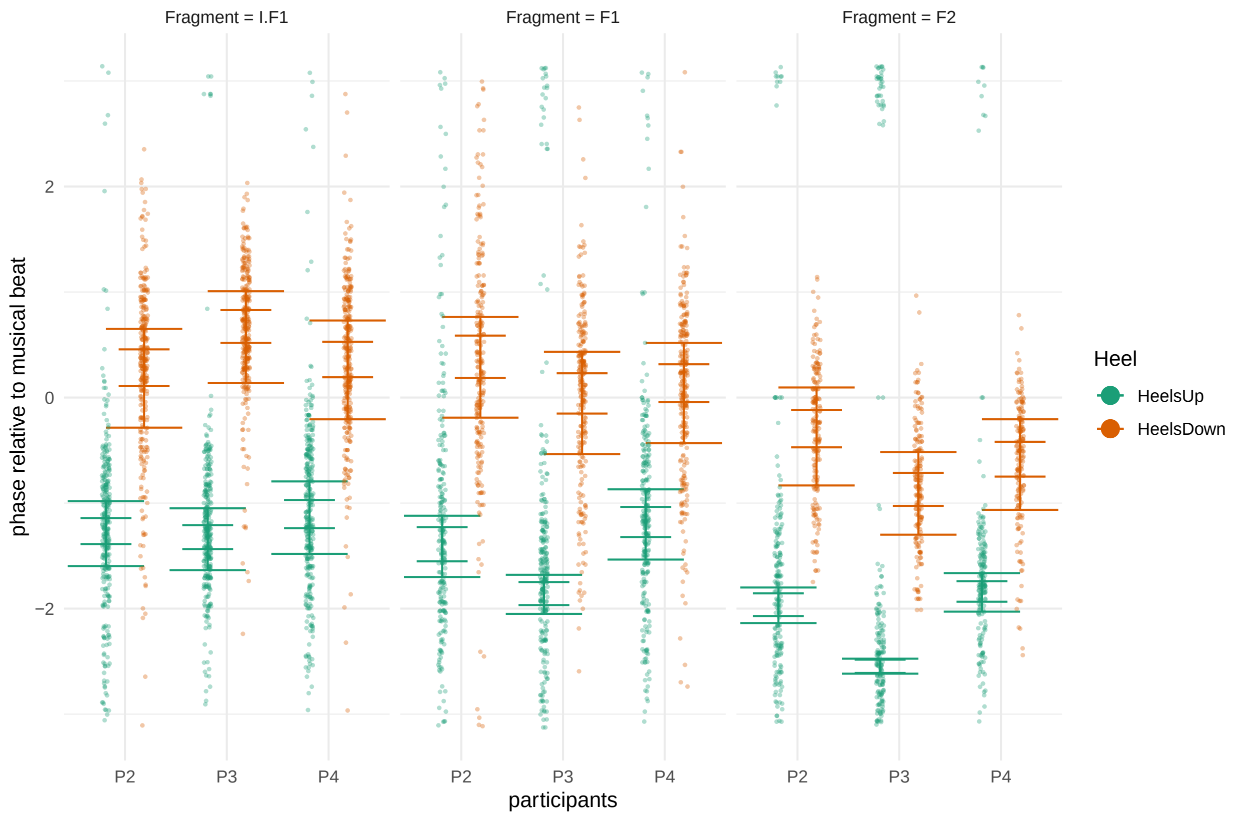

H1: Contrasts

An overview of the differences between fragments is shown in the table below. To consult the table, check the column pd>0, and then the estimate (estdiff). We can conclude that fragments I.F1, F1, F2 differ from each other, except for the HeelsUp contrast of I.F1 and F1. The HeelsDown contrast has a small effect size

of 0.23 rad. The greatest difference is found for HeelsDown between I.F1 and F2 (0.88 rad), and between F1 and F2 (0.73 rad). This supports the idea that I.F1 and F1 are only slightly different, whereas F2 is rather different from both I.F1 and F1. The estimates suggest that HeelsDown measurements occur later than the beat (positive values of the relative phase), except in F2 where it is earlier (negative values). All HeelsUp measurements occur before the beat.

The following Table shows the contrasts, see also figure 1 for further illustration.

```
## -- Attaching packages --------------------------------------- tidyverse 1.3.2 --
## v tibble  3.1.8      v dplyr   1.0.10
## v tidyr   1.2.1      v stringr 1.4.1
## v readr   2.1.3      v forcats 0.5.2
## v purrr   0.3.5
## -- Conflicts ------------------------------------------ tidyverse_conflicts() --
## x dplyr::filter() masks stats::filter()
## x dplyr::lag()    masks stats::lag()
##
## Attaching package: 'kableExtra'
##
##
## The following object is masked from 'package:dplyr':
##
##     group_rows
```

row	fragment	heel	pd>0	estdiff	lowdiff	uppdiff
1	I.F1, F1	HeelsDown, HeelsDown	100	0.21	0.12	0.32
2	F1, F2	HeelsDown, HeelsDown	100	0.77	0.65	0.87
3	I.F1, F2	HeelsDown, HeelsDown	100	0.90	0.81	1.00
4	I.F1, F1	HeelsUp, HeelsUp	100	0.34	0.24	0.43
5	F1, F2	HeelsUp, HeelsUp	100	0.66	0.55	0.76
6	I.F1, F2	HeelsUp, HeelsUp	100	0.95	0.87	1.03

Hypothesis 2: no effect of random effects

The numbers reveal that Trial 01 is an outlier with respect to the other Trials. The estimated variance of the random variable Trial is 0.13^2 = 0.01 or 1% of the total variance, which is neglectible. When looking at the details, a smallincrease in relative phase with increasing repeated measure (Trial) can be observed. However, most distributions are not or almost not different from zero, meaning that the effect of Trial is not different from the intercept.

```
## $Trialf
## , , Intercept
##
##       Estimate  Est.Error          Q2.5       Q97.5
## 01 -0.27136157 0.04899022 -0.3721373500 -0.17702433
## 02 -0.06325880 0.04657262 -0.1537323750  0.02953089
## 03 -0.10638928 0.04408593 -0.1928009500 -0.01538160
## 04  0.02112158 0.04494689 -0.0663514550  0.11165130
## 05  0.04636189 0.04812988 -0.0471962400  0.14182370
## 06 -0.06617490 0.04763086 -0.1630557000  0.02632878
```

```
## 07  0.04196090 0.04477818 -0.0446195125  0.13235942
## 08  0.05272336 0.04754344 -0.0425376650  0.14613055
## 09  0.08534576 0.04426171 -0.0006804792  0.17313540
## 10  0.01629217 0.04423756 -0.0699236600  0.10288922
## 11  0.18119158 0.04411023  0.0955706825  0.26922547
## 12  0.06491752 0.04465479 -0.0225784100  0.15309655
##
## , , kappa_Intercept
##
##       Estimate  Est.Error         Q2.5       Q97.5
## 01 -0.32275723 0.09288084 -0.521956725 -0.15927725
## 02 -0.14120371 0.08918970 -0.329236025  0.02301643
## 03  0.08449905 0.08559645 -0.088977942  0.25021590
## 04 -0.04530492 0.08770041 -0.225573925  0.11895965
## 05  0.16105221 0.11088476 -0.058886730  0.38055305
## 06 -0.31183091 0.09229294 -0.510497050 -0.14409778
## 07 -0.04683750 0.08658531 -0.224506800  0.11315522
## 08  0.21441752 0.10845115  0.002651994  0.43070155
## 09  0.04171183 0.08726292 -0.138785150  0.20397772
## 10  0.07569421 0.08593542 -0.102801075  0.24058702
## 11  0.01735755 0.08565997 -0.158280050  0.17589017
## 12 -0.02583861 0.08606871 -0.201536600  0.13613805
```

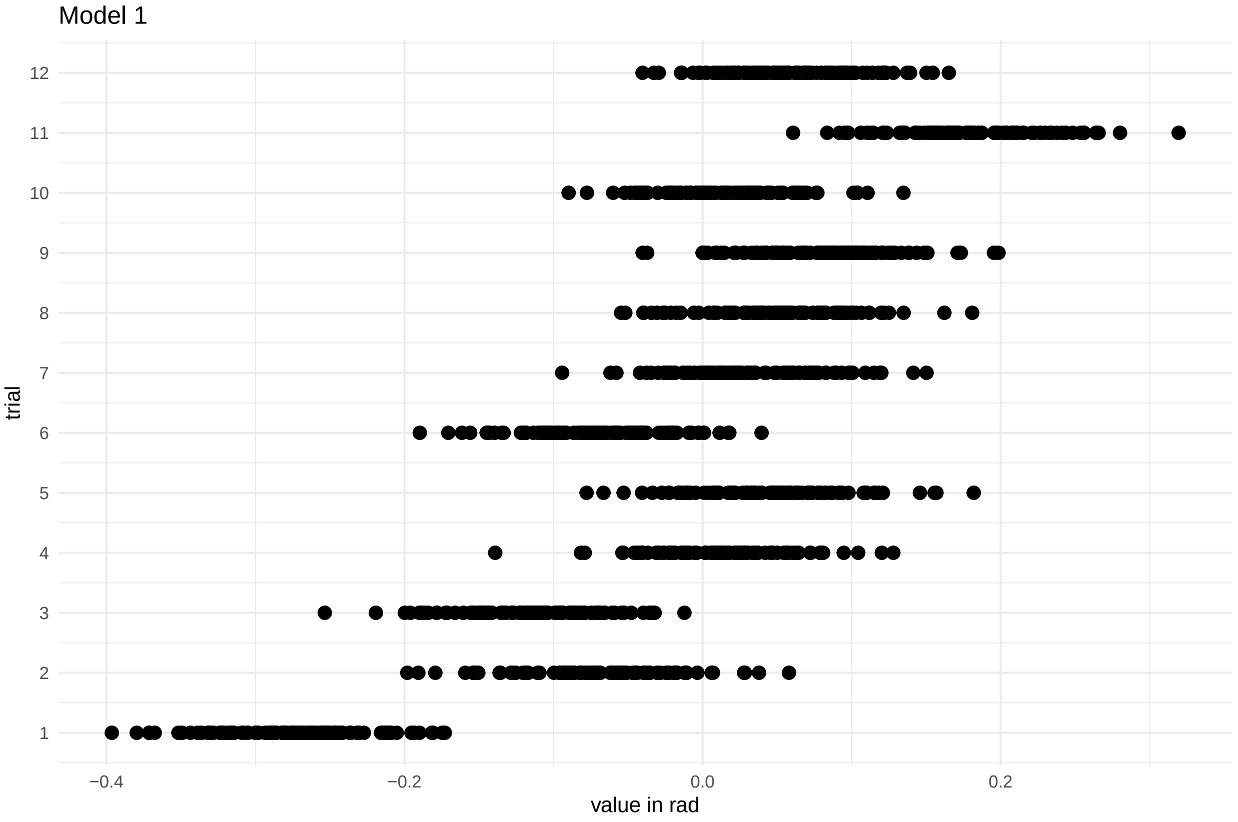

Hypothesis 3: effects of segments of between-fragments and within-fragments

H3: Diagnostics

The diagnostics of model 3 shows the posterior predictive check and the main variables.

```
## Phase1 ~ 1 + interaction(Heel, Fragment) + s(time0, by = interaction(Heel, Fragment)) + (1 | Trialf)
## kappa ~ 1 + interaction(Heel, Fragment) + s(time0, by = interaction(Heel, Fragment)) + (1 | Trialf) +
```

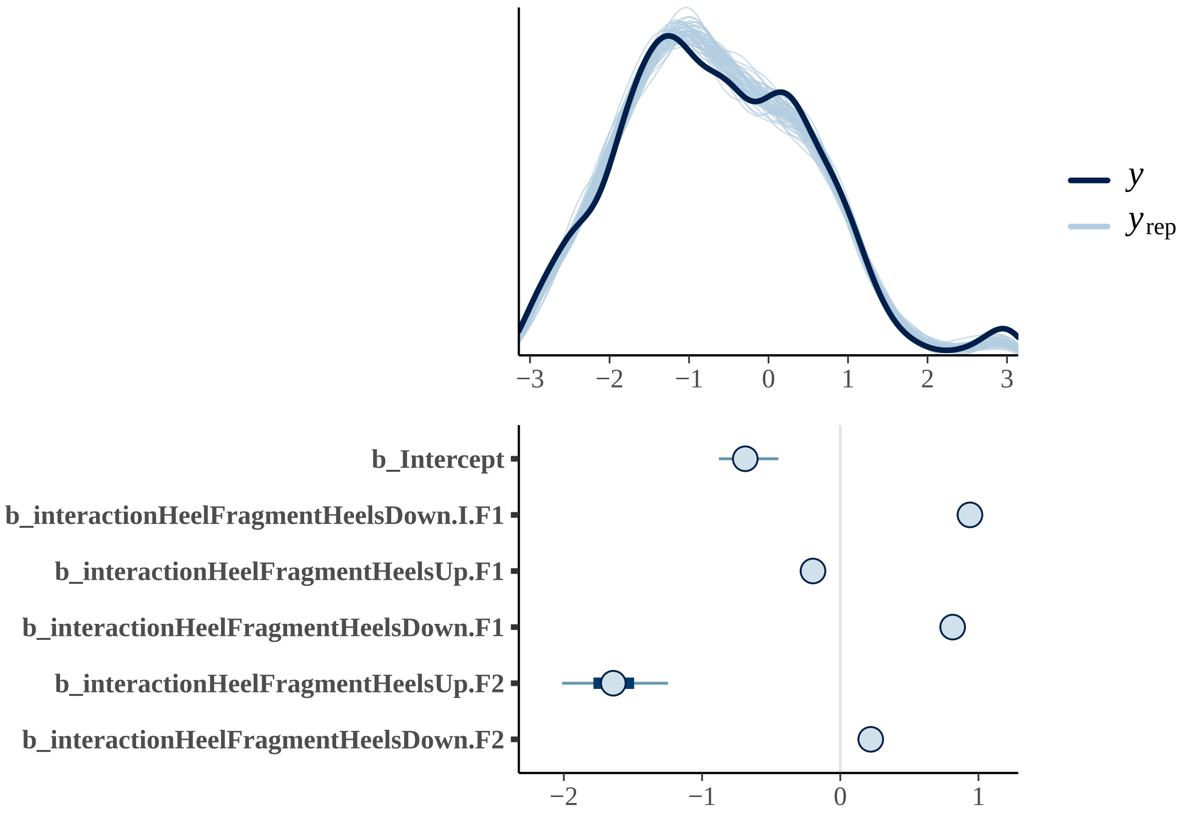


```
## Warning: There were 12 divergent transitions after warmup. Increasing
## adapt_delta above 0.8 may help. See http://mc-stan.org/misc/
## warnings.html#divergent-transitions-after-warmup

##  Family: von_mises
##   Links: mu = tan_half; kappa = log
## Formula: Phase1 ~ 1 + interaction(Heel, Fragment) + s(time0, by = interaction(Heel, Fragment)) + (1
##          kappa ~ 1 + interaction(Heel, Fragment) + s(time0, by = interaction(Heel, Fragment)) + (1 |
##    Data: Data (Number of observations: 4442)
##   Draws: 2 chains, each with iter = 8000; warmup = 3000; thin = 2;
##          total post-warmup draws = 5000
##
## Smooth Terms:
##                                                             Estimate Est.Error
```

```
## sds(stime0interactionHeelFragmentHeelsUp.I.F1_1)            2.14      0.73
## sds(stime0interactionHeelFragmentHeelsDown.I.F1_1)          1.31      0.54
## sds(stime0interactionHeelFragmentHeelsUp.F1_1)              2.88      1.13
## sds(stime0interactionHeelFragmentHeelsDown.F1_1)            1.12      0.44
## sds(stime0interactionHeelFragmentHeelsUp.F2_1)              8.69      3.68
## sds(stime0interactionHeelFragmentHeelsDown.F2_1)            1.33      0.61
## sds(kappa_stime0interactionHeelFragmentHeelsUp.I.F1_1)      3.85      1.52
## sds(kappa_stime0interactionHeelFragmentHeelsDown.I.F1_1)    3.69      1.36
## sds(kappa_stime0interactionHeelFragmentHeelsUp.F1_1)        2.49      1.11
## sds(kappa_stime0interactionHeelFragmentHeelsDown.F1_1)      2.82      1.10
## sds(kappa_stime0interactionHeelFragmentHeelsUp.F2_1)        0.75      0.78
## sds(kappa_stime0interactionHeelFragmentHeelsDown.F2_1)      0.81      0.78
##                                                          l-95% CI u-95% CI Rhat
## sds(stime0interactionHeelFragmentHeelsUp.I.F1_1)            1.08     3.87 1.00
## sds(stime0interactionHeelFragmentHeelsDown.I.F1_1)          0.58     2.65 1.00
## sds(stime0interactionHeelFragmentHeelsUp.F1_1)              1.26     5.67 1.00
## sds(stime0interactionHeelFragmentHeelsDown.F1_1)            0.53     2.17 1.00
## sds(stime0interactionHeelFragmentHeelsUp.F2_1)              4.26    17.91 1.00
## sds(stime0interactionHeelFragmentHeelsDown.F2_1)            0.50     2.85 1.00
## sds(kappa_stime0interactionHeelFragmentHeelsUp.I.F1_1)      1.72     7.63 1.00
## sds(kappa_stime0interactionHeelFragmentHeelsDown.I.F1_1)    1.78     7.00 1.00
## sds(kappa_stime0interactionHeelFragmentHeelsUp.F1_1)        1.08     5.35 1.00
## sds(kappa_stime0interactionHeelFragmentHeelsDown.F1_1)      1.35     5.57 1.00
## sds(kappa_stime0interactionHeelFragmentHeelsUp.F2_1)        0.02     2.81 1.00
## sds(kappa_stime0interactionHeelFragmentHeelsDown.F2_1)      0.03     2.90 1.00
##                                                          Bulk_ESS Tail_ESS
## sds(stime0interactionHeelFragmentHeelsUp.I.F1_1)            2571     3277
## sds(stime0interactionHeelFragmentHeelsDown.I.F1_1)          2686     3266
## sds(stime0interactionHeelFragmentHeelsUp.F1_1)              2419     2970
## sds(stime0interactionHeelFragmentHeelsDown.F1_1)            3106     3841
## sds(stime0interactionHeelFragmentHeelsUp.F2_1)              2493     3009
## sds(stime0interactionHeelFragmentHeelsDown.F2_1)            2460     3289
## sds(kappa_stime0interactionHeelFragmentHeelsUp.I.F1_1)      2916     3392
## sds(kappa_stime0interactionHeelFragmentHeelsDown.I.F1_1)    3190     3874
## sds(kappa_stime0interactionHeelFragmentHeelsUp.F1_1)        3054     3647
## sds(kappa_stime0interactionHeelFragmentHeelsDown.F1_1)      3590     4419
## sds(kappa_stime0interactionHeelFragmentHeelsUp.F2_1)        3229     3024
## sds(kappa_stime0interactionHeelFragmentHeelsDown.F2_1)      3223     3369
##
## Group-Level Effects:
## ~Participant (Number of levels: 3)
##                     Estimate Est.Error l-95% CI u-95% CI Rhat Bulk_ESS Tail_ESS
## sd(Intercept)           0.20      0.28     0.03     0.97 1.00     1999     2058
## sd(kappa_Intercept)     3.21      1.62     0.81     7.29 1.00     2855     1896
##
## ~Trialf (Number of levels: 12)
##                     Estimate Est.Error l-95% CI u-95% CI Rhat Bulk_ESS Tail_ESS
## sd(Intercept)           0.14      0.04     0.09     0.24 1.00     2373     3599
## sd(kappa_Intercept)     0.33      0.09     0.20     0.54 1.00     2645     3620
##
## Population-Level Effects:
##                                                      Estimate Est.Error
## Intercept                                               -0.68      0.16
## kappa_Intercept                                          4.24      0.95
```

```
## interactionHeelFragmentHeelsDown.I.F1                  0.94      0.02
## interactionHeelFragmentHeelsUp.F1                     -0.20      0.03
## interactionHeelFragmentHeelsDown.F1                    0.81      0.02
## interactionHeelFragmentHeelsUp.F2                     -1.63      0.24
## interactionHeelFragmentHeelsDown.F2                    0.22      0.05
## kappa_interactionHeelFragmentHeelsDown.I.F1           -0.12      0.07
## kappa_interactionHeelFragmentHeelsUp.F1               -0.28      0.07
## kappa_interactionHeelFragmentHeelsDown.F1             -0.19      0.08
## kappa_interactionHeelFragmentHeelsUp.F2                0.01      0.09
## kappa_interactionHeelFragmentHeelsDown.F2              0.29      0.11
## stime0:interactionHeelFragmentHeelsUp.I.F1_1           0.17      0.49
## stime0:interactionHeelFragmentHeelsDown.I.F1_1         0.31      0.48
## stime0:interactionHeelFragmentHeelsUp.F1_1            -0.17      0.50
## stime0:interactionHeelFragmentHeelsDown.F1_1          -0.16      0.47
## stime0:interactionHeelFragmentHeelsUp.F2_1            -0.02      0.50
## stime0:interactionHeelFragmentHeelsDown.F2_1          -0.14      0.50
## kappa_stime0:interactionHeelFragmentHeelsUp.I.F1_1    17.60      5.20
## kappa_stime0:interactionHeelFragmentHeelsDown.I.F1_1  17.08      5.59
## kappa_stime0:interactionHeelFragmentHeelsUp.F1_1       7.04      4.64
## kappa_stime0:interactionHeelFragmentHeelsDown.F1_1    10.40      5.01
## kappa_stime0:interactionHeelFragmentHeelsUp.F2_1       1.25      2.95
## kappa_stime0:interactionHeelFragmentHeelsDown.F2_1     0.33      2.95
##                                                     l-95% CI u-95% CI Rhat
## Intercept                                             -1.00    -0.32 1.00
## kappa_Intercept                                        2.13     5.98 1.00
## interactionHeelFragmentHeelsDown.I.F1                  0.90     0.98 1.00
## interactionHeelFragmentHeelsUp.F1                     -0.26    -0.13 1.00
## interactionHeelFragmentHeelsDown.F1                    0.77     0.86 1.00
## interactionHeelFragmentHeelsUp.F2                     -2.09    -1.14 1.00
## interactionHeelFragmentHeelsDown.F2                    0.13     0.32 1.00
## kappa_interactionHeelFragmentHeelsDown.I.F1           -0.25     0.02 1.00
## kappa_interactionHeelFragmentHeelsUp.F1               -0.43    -0.14 1.00
## kappa_interactionHeelFragmentHeelsDown.F1             -0.34    -0.04 1.00
## kappa_interactionHeelFragmentHeelsUp.F2               -0.16     0.20 1.00
## kappa_interactionHeelFragmentHeelsDown.F2              0.08     0.51 1.00
## stime0:interactionHeelFragmentHeelsUp.I.F1_1          -0.77     1.13 1.00
## stime0:interactionHeelFragmentHeelsDown.I.F1_1        -0.65     1.25 1.00
## stime0:interactionHeelFragmentHeelsUp.F1_1            -1.12     0.81 1.00
## stime0:interactionHeelFragmentHeelsDown.F1_1          -1.09     0.78 1.00
## stime0:interactionHeelFragmentHeelsUp.F2_1            -1.02     0.96 1.00
## stime0:interactionHeelFragmentHeelsDown.F2_1          -1.10     0.84 1.00
## kappa_stime0:interactionHeelFragmentHeelsUp.I.F1_1     8.84    29.03 1.00
## kappa_stime0:interactionHeelFragmentHeelsDown.I.F1_1   7.56    29.62 1.00
## kappa_stime0:interactionHeelFragmentHeelsUp.F1_1      -0.46    18.23 1.00
## kappa_stime0:interactionHeelFragmentHeelsDown.F1_1     1.49    21.43 1.00
## kappa_stime0:interactionHeelFragmentHeelsUp.F2_1      -3.37     8.51 1.00
## kappa_stime0:interactionHeelFragmentHeelsDown.F2_1    -5.86     6.66 1.00
##                                                     Bulk_ESS Tail_ESS
## Intercept                                              2273     1997
## kappa_Intercept                                        3117     1905
## interactionHeelFragmentHeelsDown.I.F1                  4070     4502
## interactionHeelFragmentHeelsUp.F1                      3564     3734
## interactionHeelFragmentHeelsDown.F1                    3858     4073
## interactionHeelFragmentHeelsUp.F2                      2734     2983
```

```
## interactionHeelFragmentHeelsDown.F2                       3424     3461
## kappa_interactionHeelFragmentHeelsDown.I.F1               3776     4494
## kappa_interactionHeelFragmentHeelsUp.F1                   3862     4299
## kappa_interactionHeelFragmentHeelsDown.F1                 3951     4199
## kappa_interactionHeelFragmentHeelsUp.F2                   3199     3385
## kappa_interactionHeelFragmentHeelsDown.F2                 3899     3687
## stime0:interactionHeelFragmentHeelsUp.I.F1_1              3990     3994
## stime0:interactionHeelFragmentHeelsDown.I.F1_1            3871     3993
## stime0:interactionHeelFragmentHeelsUp.F1_1                4019     3988
## stime0:interactionHeelFragmentHeelsDown.F1_1              4437     3855
## stime0:interactionHeelFragmentHeelsUp.F2_1                3965     3752
## stime0:interactionHeelFragmentHeelsDown.F2_1              3494     3607
## kappa_stime0:interactionHeelFragmentHeelsUp.I.F1_1        3220     3624
## kappa_stime0:interactionHeelFragmentHeelsDown.I.F1_1      4131     4212
## kappa_stime0:interactionHeelFragmentHeelsUp.F1_1          3103     3609
## kappa_stime0:interactionHeelFragmentHeelsDown.F1_1        3985     4364
## kappa_stime0:interactionHeelFragmentHeelsUp.F2_1          3206     2678
## kappa_stime0:interactionHeelFragmentHeelsDown.F2_1        3822     3283
##
## Draws were sampled using sample(hmc). For each parameter, Bulk_ESS
## and Tail_ESS are effective sample size measures, and Rhat is the potential
## scale reduction factor on split chains (at convergence, Rhat = 1).
```

H3: Posterior predictive distributions (retrodiction)

Gray regions indicate the CI with 95% probablity mass in favour of the expected value of the posterior predictive distribution, excluding the group-effect of `Trial`.

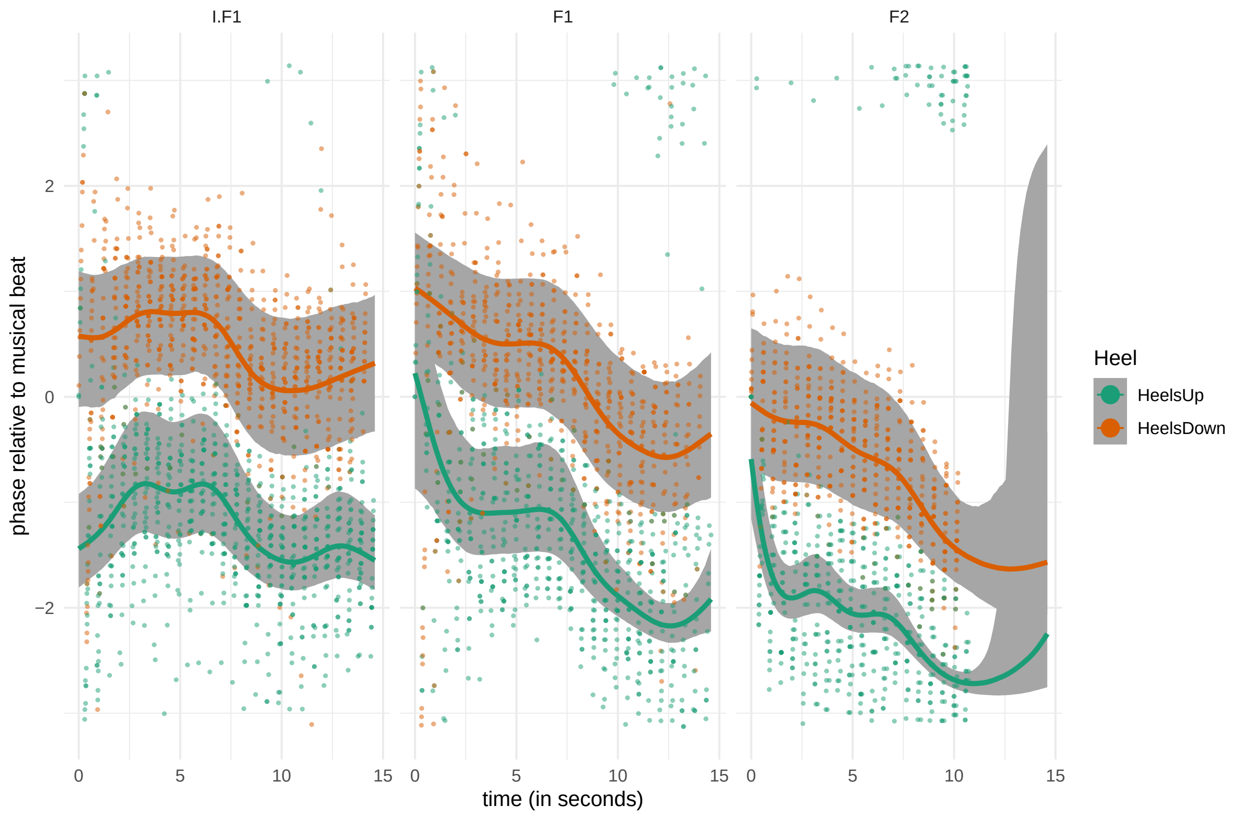

H3: Contrasts

The table shows a summary of the contrasts calculated for different time segments. Based on the time annotated music score, we consider the following contrast types:

- Between-repeats 1-3: this is a contrast of a segment from 0 to 4.25 seconds of phrase 1 and a segment 2 from 9 to 13.25 seconds of phrase 3.
- Between-repeats 2-4: this is a contrast of a segment from 4.26 to 6 seconds of phrase 2 and a segment 2 from 13.26 to 15 seconds of phrase 4.
- Within-section 1: this is a contrast of a segment from 2.51 to 4.25 seconds of phrase 1 and a segment from 4.26 to 6 seconds of phrase 2.
- Within-section 2: this is a contrast of a segment from 11.51 to 13.25 seconds of phrase 3 and a segment from 13.26 to 15 seconds of phrase 4.
- Between-fragments: this contrast omits the first 2 seconds of the fragment, avoiding large variability in data due to the beginning of the dance.

Phrases are defined in the annotate score: phrase 1 and 2 define segment 1, and phrase 2 and 4 define segment 2, except for F2 where we have no phrase 4. Repeated phrases are also called repeats. To consult the table, check the column pd>0 to start with, then check what kind of contrast is considered. Values above 95% passed the test of having 95% of the prob mass above zero, meaning that the contrast shows strong evidence for the hypothesis that seg1 has higher relative phase than seg2. How high? Check estdiff. Check also for reverse evidence, see row 3 and 19. Overall, we see evidence for difference among between-repeats

(i.e. comparison of two segments having similar musical context), and no-difference among within-phrases (i.e. comparison of two segments within a phrase). In row 4 and 19, it appears that seg2 is actually somewhat higher than seg1 in F1. It might be due to a preparation for the subsequent musical context.

row	contrast	fragment	heel	pd>0	estdiff	lowdiff	uppdiff
1	C1 between-repeats 1-3	I.F1, I.F1	HeelsDown, HeelsDown	100.00	0.53	0.39	0.66
2	C2 between-repeats 2-4	I.F1, I.F1	HeelsDown, HeelsDown	100.00	0.59	0.47	0.69
3	C3 within-section 1	I.F1, I.F1	HeelsDown, HeelsDown	10.68	-0.05	-0.13	0.02
4	C4 within-section 2	I.F1, I.F1	HeelsDown, HeelsDown	1.34	-0.11	-0.19	-0.02
5	C1 between-repeats 1-3	F1, F1	HeelsDown, HeelsDown	100.00	1.05	0.87	1.21
6	C2 between-repeats 2-4	F1, F1	HeelsDown, HeelsDown	100.00	1.00	0.86	1.17
7	C3 within-section 1	F1, F1	HeelsDown, HeelsDown	98.64	0.12	0.02	0.25
8	C4 within-section 2	F1, F1	HeelsDown, HeelsDown	44.64	-0.03	-0.13	0.10
9	C1 between-repeats 1-3	F2, F2	HeelsDown, HeelsDown	100.00	1.05	0.94	1.15
10	C3 within-section 1	F2, F2	HeelsDown, HeelsDown	100.00	0.19	0.09	0.29
11	Contrast between-fragments	F1, F2	HeelsDown, HeelsDown	100.00	0.81	0.75	0.87
12	C1 between-repeats 1-3	I.F1, I.F1	HeelsUp, HeelsUp	100.00	0.42	0.30	0.55
13	C2 between-repeats 2-4	I.F1, I.F1	HeelsUp, HeelsUp	100.00	0.58	0.47	0.68
14	C3 within-section 1	I.F1, I.F1	HeelsUp, HeelsUp	49.38	-0.01	-0.10	0.10
15	C4 within-section 2	I.F1, I.F1	HeelsUp, HeelsUp	29.32	-0.02	-0.10	0.06
16	C1 between-repeats 1-3	F1, F1	HeelsUp, HeelsUp	100.00	1.18	0.96	1.40
17	C2 between-repeats 2-4	F1, F1	HeelsUp, HeelsUp	100.00	1.01	0.83	1.16
18	C3 within-section 1	F1, F1	HeelsUp, HeelsUp	86.38	0.06	-0.05	0.17
19	C4 within-section 2	F1, F1	HeelsUp, HeelsUp	50.00	0.01	-0.13	0.11
20	C1 between-repeats 1-3	F2, F2	HeelsUp, HeelsUp	100.00	0.98	0.86	1.07
21	C3 within-section 1	F2, F2	HeelsUp, HeelsUp	99.52	0.17	0.03	0.31
22	Contrast between-fragments	F1, F2	HeelsUp, HeelsUp	100.00	0.75	0.68	0.81

Contrast in I.F1 HeelsDown

See figure 2.

Contrast in F1 HeelsDown

See figure 3.

Contrast in F2 HeelsDown

Owing to a shorter F2 we calculated only contrast 1 and contrast 3. See figure 4.

Contrast between Fragments HeelsDown

The contrast we are looking at here is: seg1 [2,13.5] <-> seg2 [2,11] = contrast between Fragment 1 and Fragment 2, discarting the first 2 seconds

See figure 5.

Contrast in I.F1 HeelsUp

See figure 6.

Contrast in F1 HeelsUp

See figure 7.

Contrast in F2 HeelsUp

Owing to a shorter F2 we calculated only contrast 1 and contrast 3. See figure 8.

Contrast between Fragments HeelsUp

The contrast we are looking at here is F1.seg[2,13.5] <-> F2.seg[2,11], discarting the first 2 seconds. See figure 9.

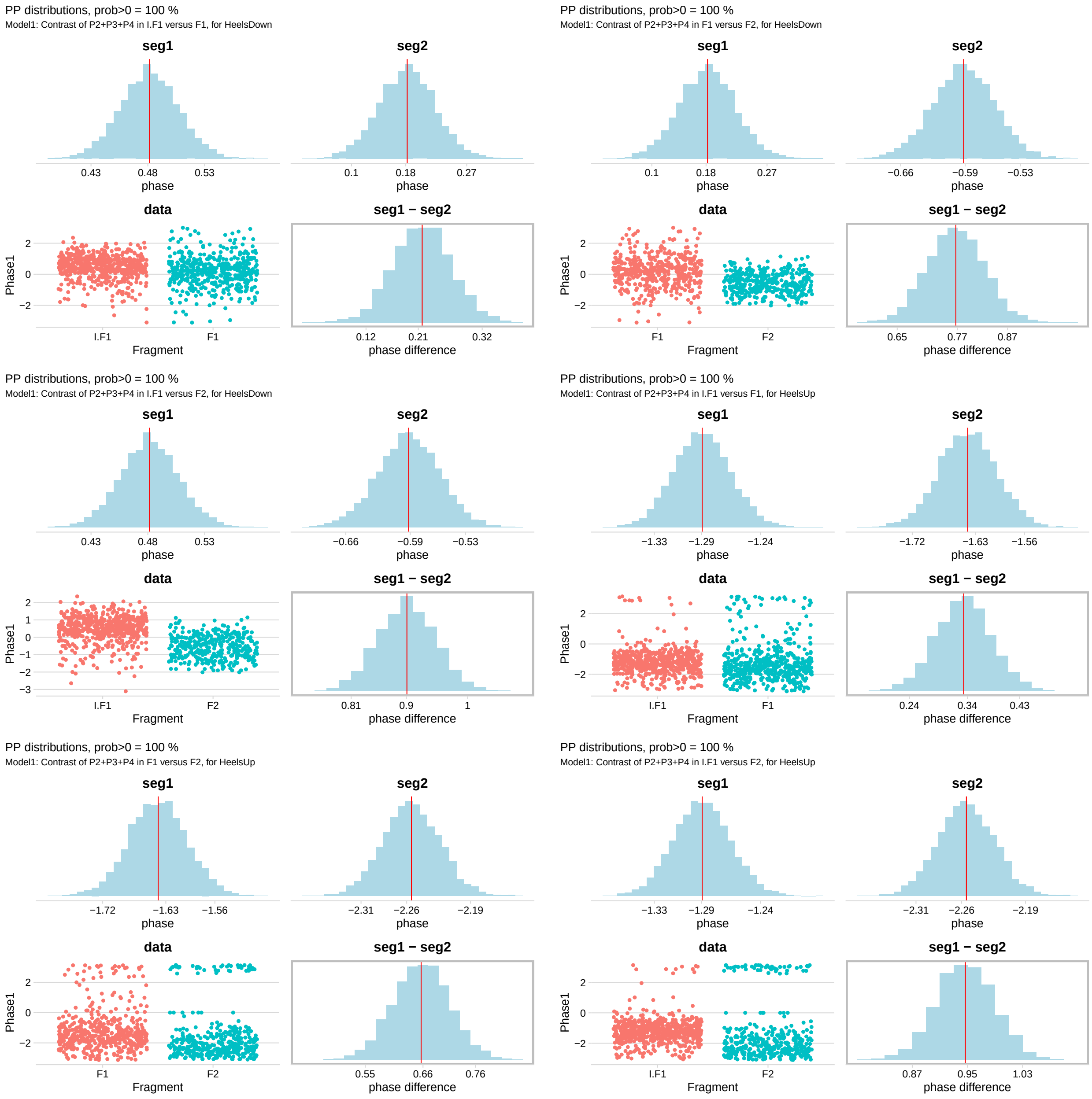

Figure 1: Contrasts among Fragments (1-3 HeelsDown, 4-6 HeelsUp)

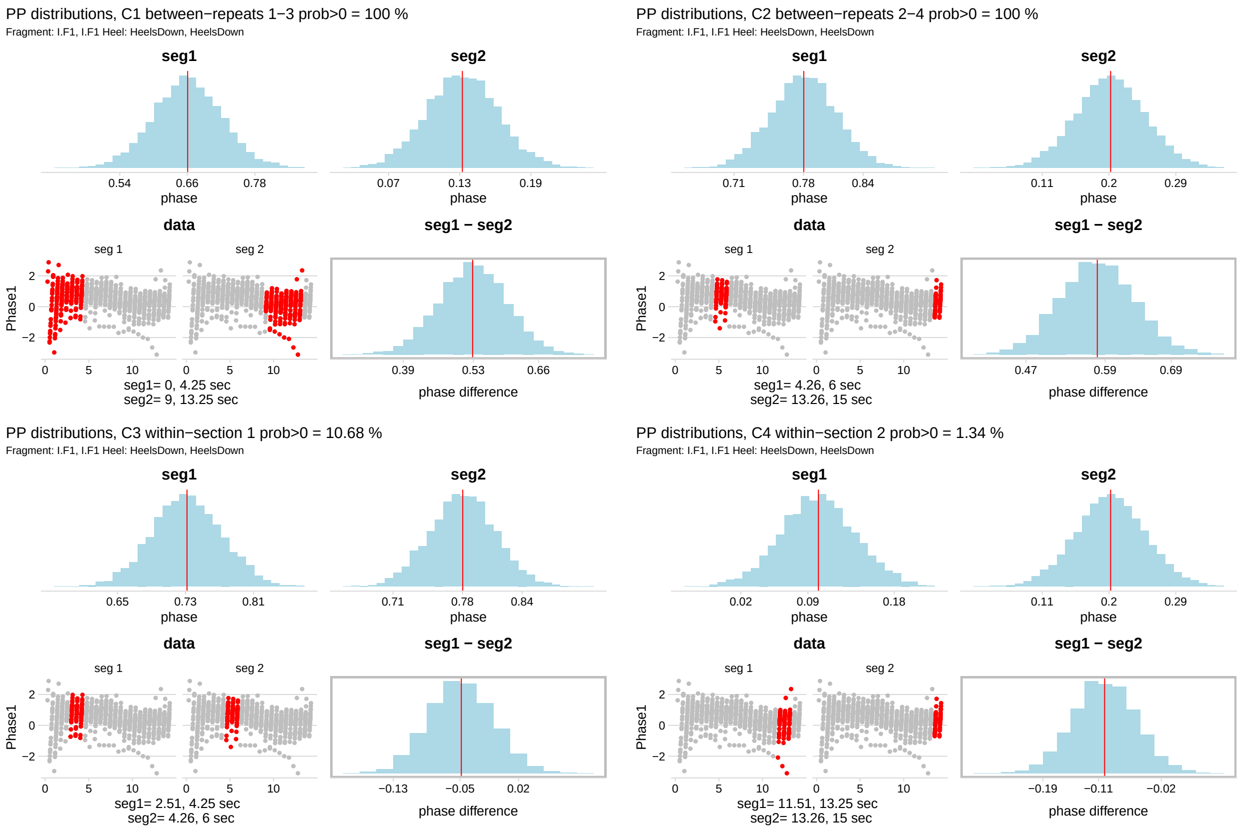

Figure 2: Contrast in I.F1, within-fragment

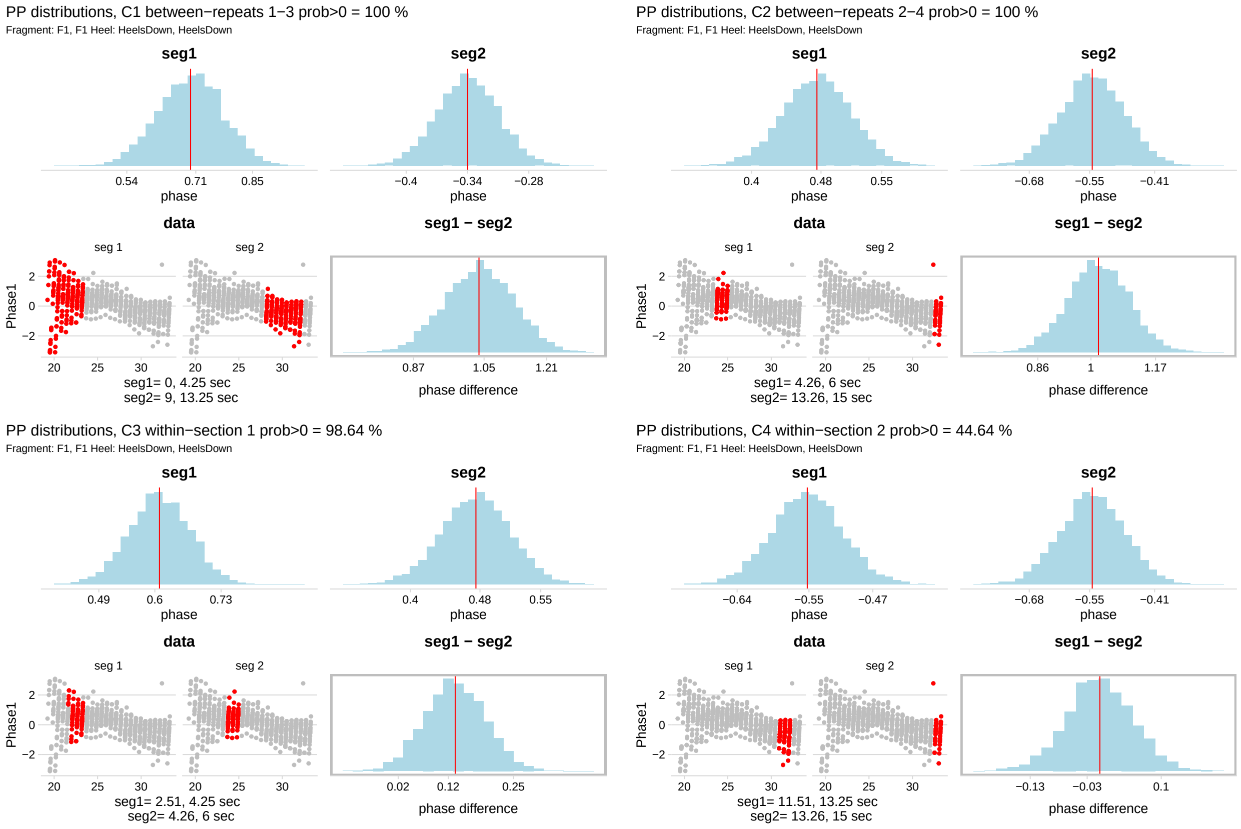

Figure 3: Contrast in F1, within-fragment

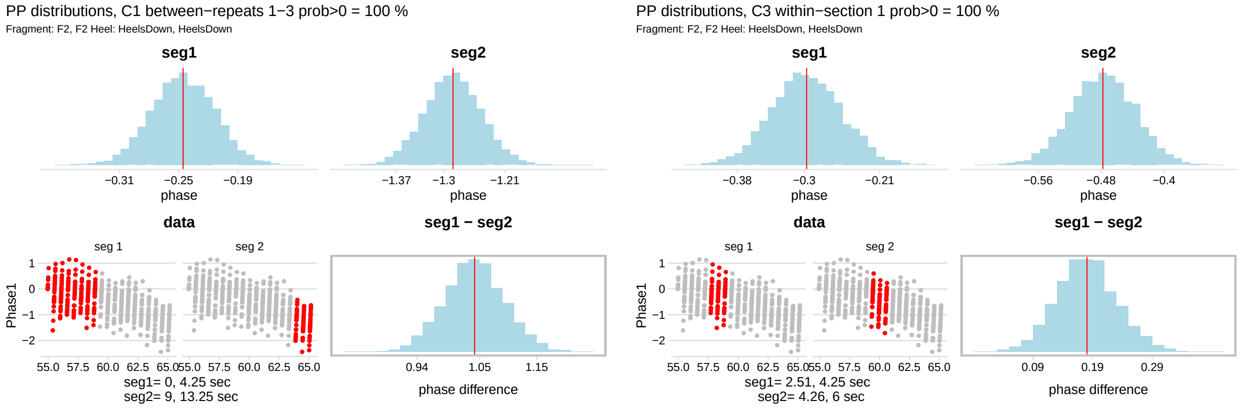

Figure 4: Contrast in F2, between and within fragments

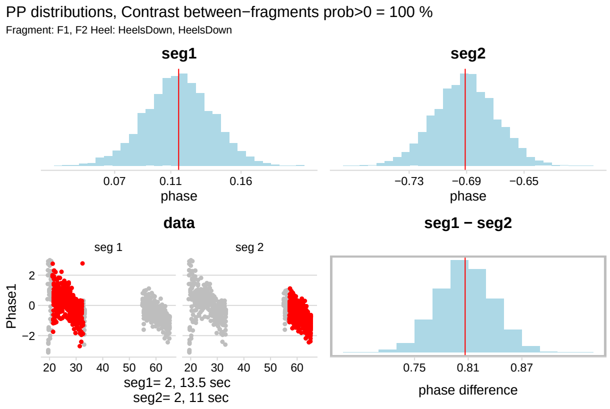

Figure 5: Contrast of F1 versus F2

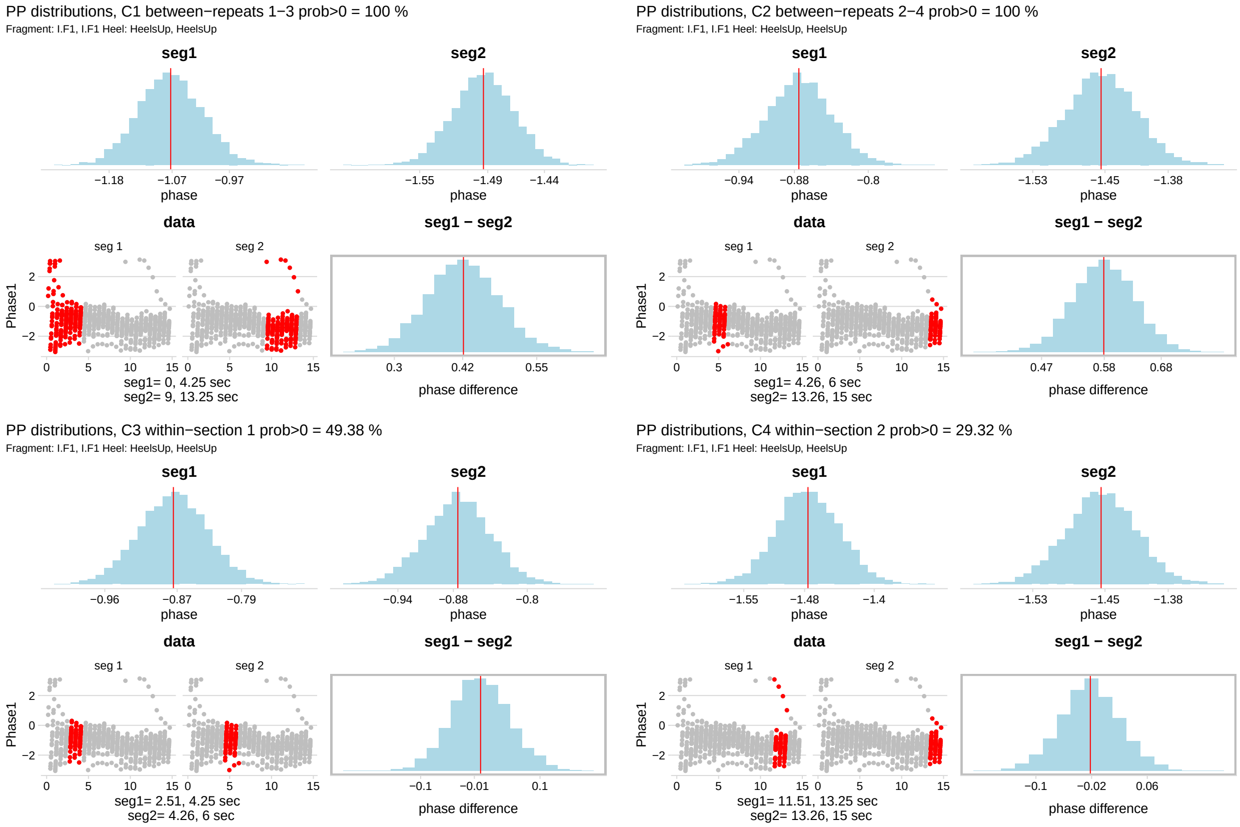

Figure 6: Contrast in I.F1, within phrases

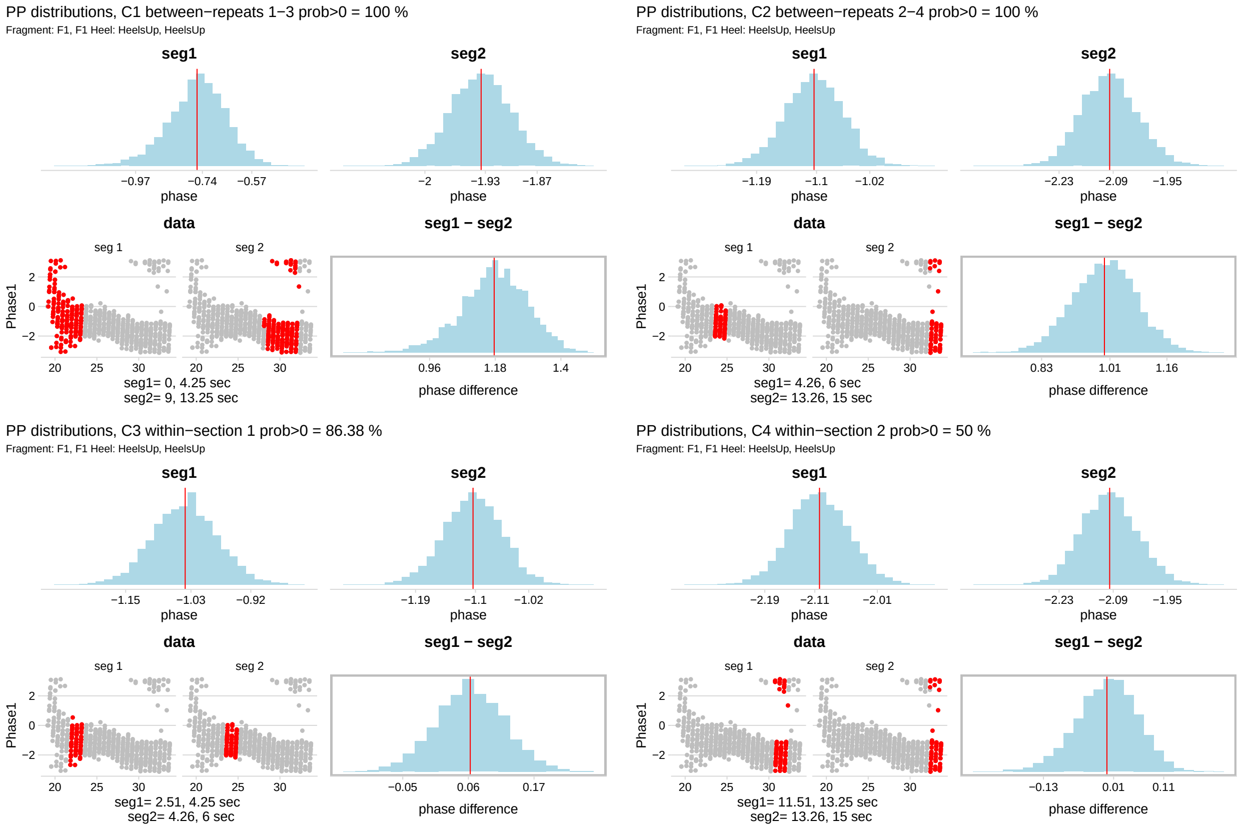

Figure 7: Contrast in F1, within phrases

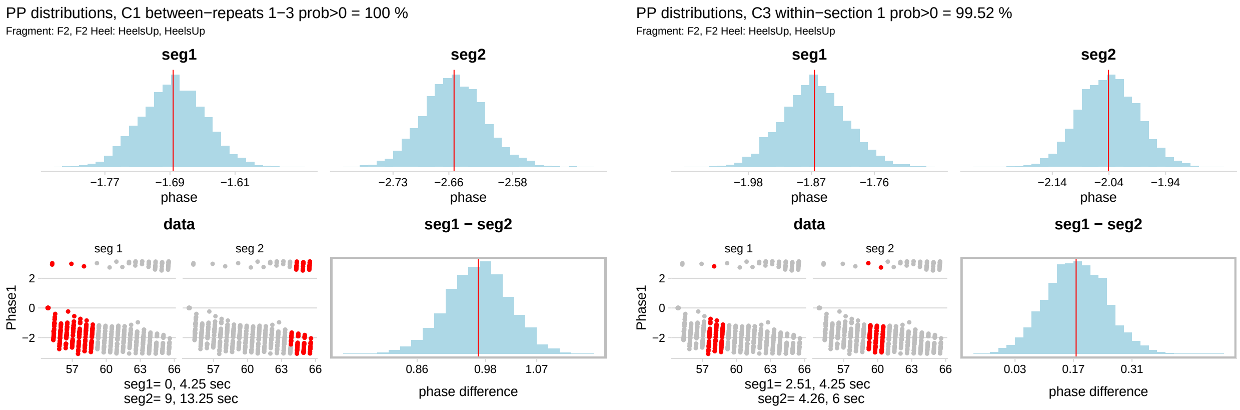

Figure 8: Contrast in F2, between and within phrases

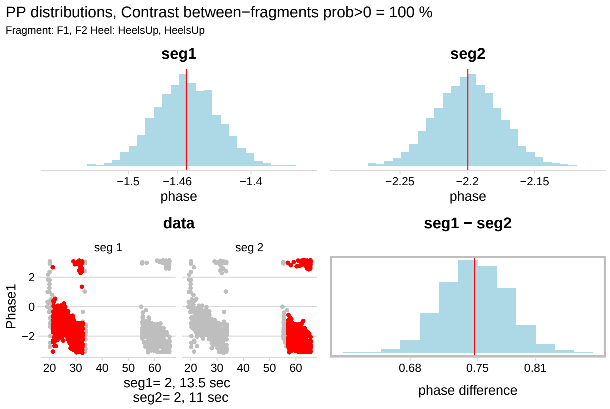

Figure 9: Contrast of F1 versus F2
