# Supplementary material for: Influence of musical context on sensorimotor synchronization in classical ballet solo dance
Source: PLoS One. 2023 Apr 18;18(4):e0284387. doi: 10.1371/journal.pone.0284387 (PMC10112816; doi:10.1371/journal.pone.0284387)
Supplement: S2 Protocol — (PDF) [file pone.0284387.s005.pdf]

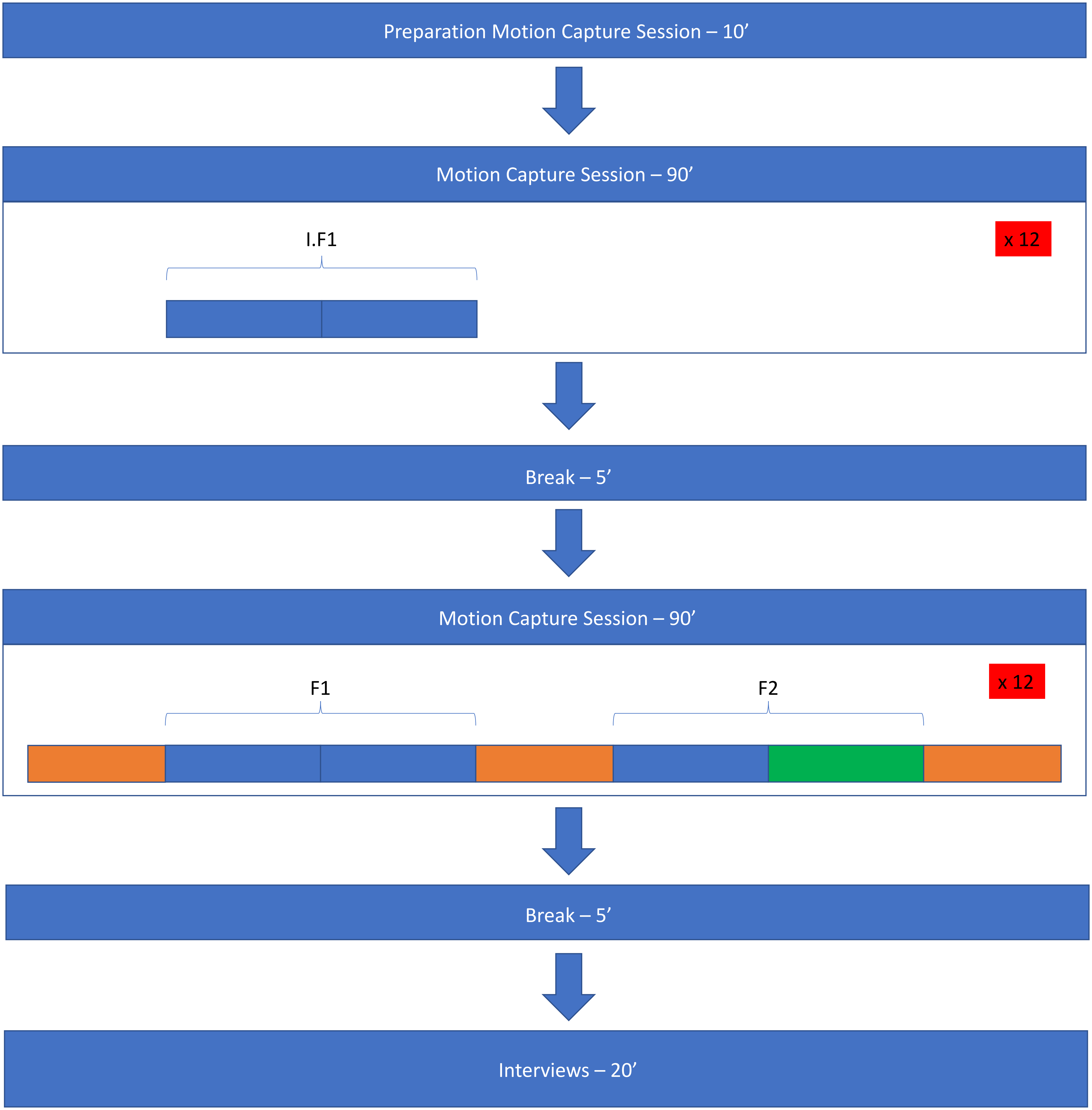
Preparation Motion Capture Session – 10’
Motion Capture Session – 90’
I.F1
x 12
Break – 5’
Motion Capture Session – 90’
F1
F2
x 12
Break – 5’
Interviews – 20’

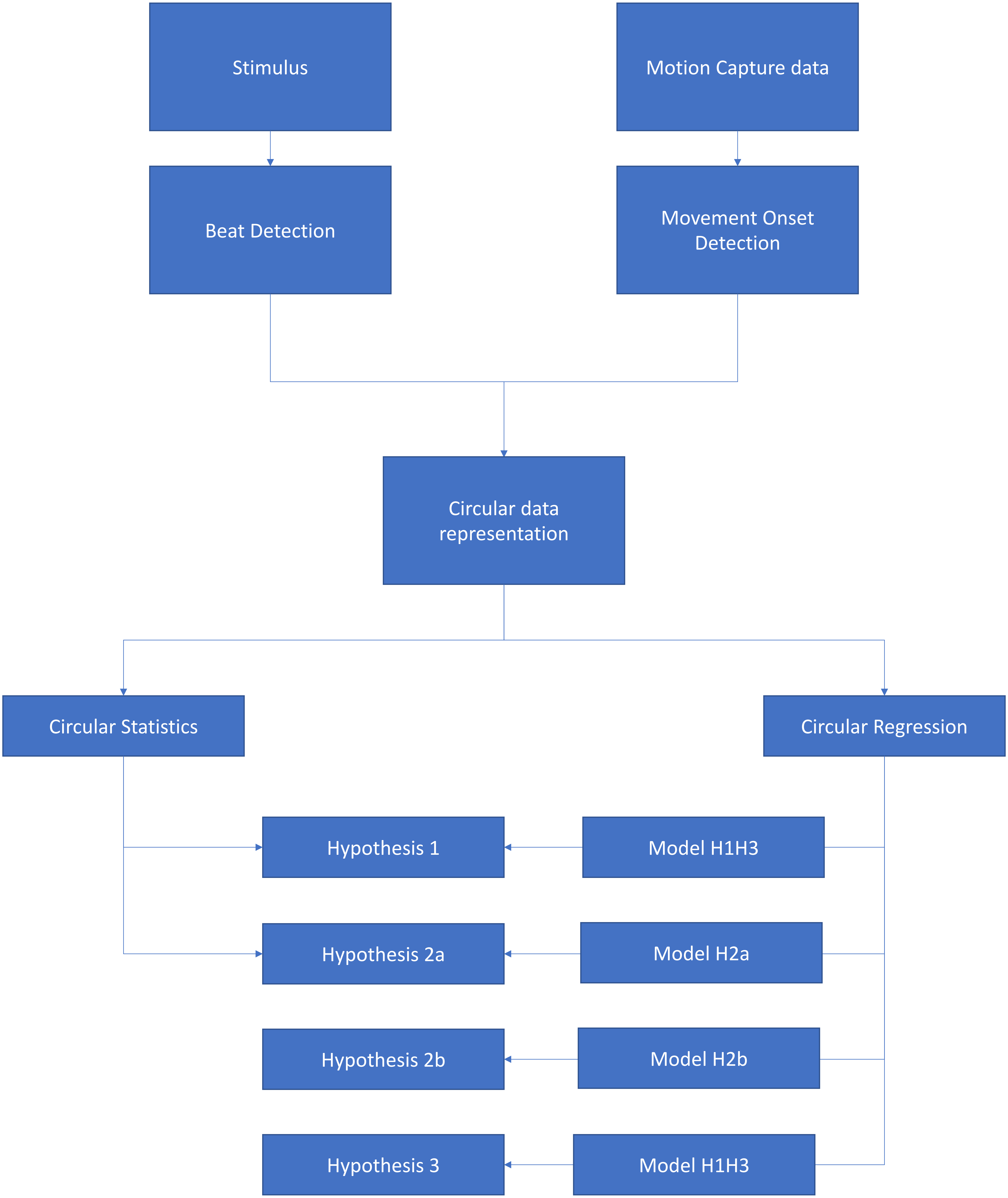
Stimulus
Motion Capture data
Beat Detection
Movement Onset Detection
Circular data representation
Circular Statistics
Circular Regression
Hypothesis 1
Model H1H3
Hypothesis 2a
Model H2a
Hypothesis 2b
Model H2b
Hypothesis 3
Model H1H3
